# Anatomy of hierarchy: Feedforward and feedback pathways in macaque visual cortex

**DOI:** 10.1002/cne.23458

**Published:** 2013-11-26

**Authors:** Nikola T Markov, Julien Vezoli, Pascal Chameau, Arnaud Falchier, René Quilodran, Cyril Huissoud, Camille Lamy, Pierre Misery, Pascale Giroud, Shimon Ullman, Pascal Barone, Colette Dehay, Kenneth Knoblauch, Henry Kennedy

**Affiliations:** 1 Stem Cell and Brain Research InstituteINSERM U846, 69500, Bron, France; 2 Université de Lyon, Université Lyon I69003, Lyon, France; 3 Department of Neurobiology, Yale University School of MedicineNew Haven, Connecticut, 06520-8001, USA; 4 Department of Computer Science, Weizmann Institute of ScienceRehovot, 76100, Israel

**Keywords:** neocortex, monkey, retrograde tracing, cell morphology

## Abstract

The laminar location of the cell bodies and terminals of interareal connections determines the hierarchical structural organization of the cortex and has been intensively studied. However, we still have only a rudimentary understanding of the connectional principles of feedforward (FF) and feedback (FB) pathways. Quantitative analysis of retrograde tracers was used to extend the notion that the laminar distribution of neurons interconnecting visual areas provides an index of hierarchical distance (percentage of supragranular labeled neurons [SLN]). We show that: 1) SLN values constrain models of cortical hierarchy, revealing previously unsuspected areal relations; 2) SLN reflects the operation of a combinatorial distance rule acting differentially on sets of connections between areas; 3) Supragranular layers contain highly segregated bottom-up and top-down streams, both of which exhibit point-to-point connectivity. This contrasts with the infragranular layers, which contain diffuse bottom-up and top-down streams; 4) Cell filling of the parent neurons of FF and FB pathways provides further evidence of compartmentalization; 5) FF pathways have higher weights, cross fewer hierarchical levels, and are less numerous than FB pathways. Taken together, the present results suggest that cortical hierarchies are built from supra- and infragranular counterstreams. This compartmentalized dual counterstream organization allows point-to-point connectivity in both bottom-up and top-down directions.

Our knowledge of how interareal connections are integrated into the local connectivity of the cortex is derived from numerous high-resolution tract-tracing experiments published over the last 40 years and, more recently, from multilaminar electrophysiological recording. This has provided the bedrock for present-day models of cortical hierarchy. Hence, it is now possible to construct large-scale computational models, incorporating hierarchical integration of interareal connections into the local circuit of the cortex (Bastos et al., 2012), and thereby link up the concepts of cortical hierarchy, Bayesian inference, and the canonical circuit (Douglas and Martin, 2007; Friston, 2010; Markov and Kennedy, 2013). As will be shown in the Discussion below, recent developments in cortical physiology have given functional insight into interareal connectivity and the concept of cortical hierarchy. Given the role of oscillatory coherence in communication (Fries, 2005), the recent demonstration that there are laminar differences in oscillatory coherence (Buffalo et al., 2011) makes the question of the laminar regularities of interareal connectivity (i.e., cortical hierarchy) highly topical. The anatomical findings of the current study suggest novel constraints on these regularities. As some of the present results echo earlier findings, we shall first review the classical literature on cortical hierarchy.

There are strong regularities in the cortical projections of early visual areas: rostral directed pathways are found to originate largely from supragranular layer neurons, and terminate in their target areas in layer 4 (Cragg, 1969; Spatz et al., 1970; Lund et al., 1975; Martinez-Millan and Hollander, 1975; Van Essen and Zeki, 1978; Wong-Riley, 1978; Rockland and Pandya, 1979), while caudal directed pathways mostly originate from infragranular layers and terminate outside of layer 4 in their target areas (Kuypers et al., 1965; Tigges et al., 1973; Kaas and Lin, 1977; Wong-Riley, 1978; Kennedy and Bullier, 1985) (Fig. 1). By analogy to the pathways linking thalamus and cortex, these findings suggest that rostral directed connections are feedforward (FF) pathways channeling information from lower to higher-order areas, while caudal directed pathways are feedback (FB) pathways (Rockland and Pandya, 1979).

**Figure 1:**
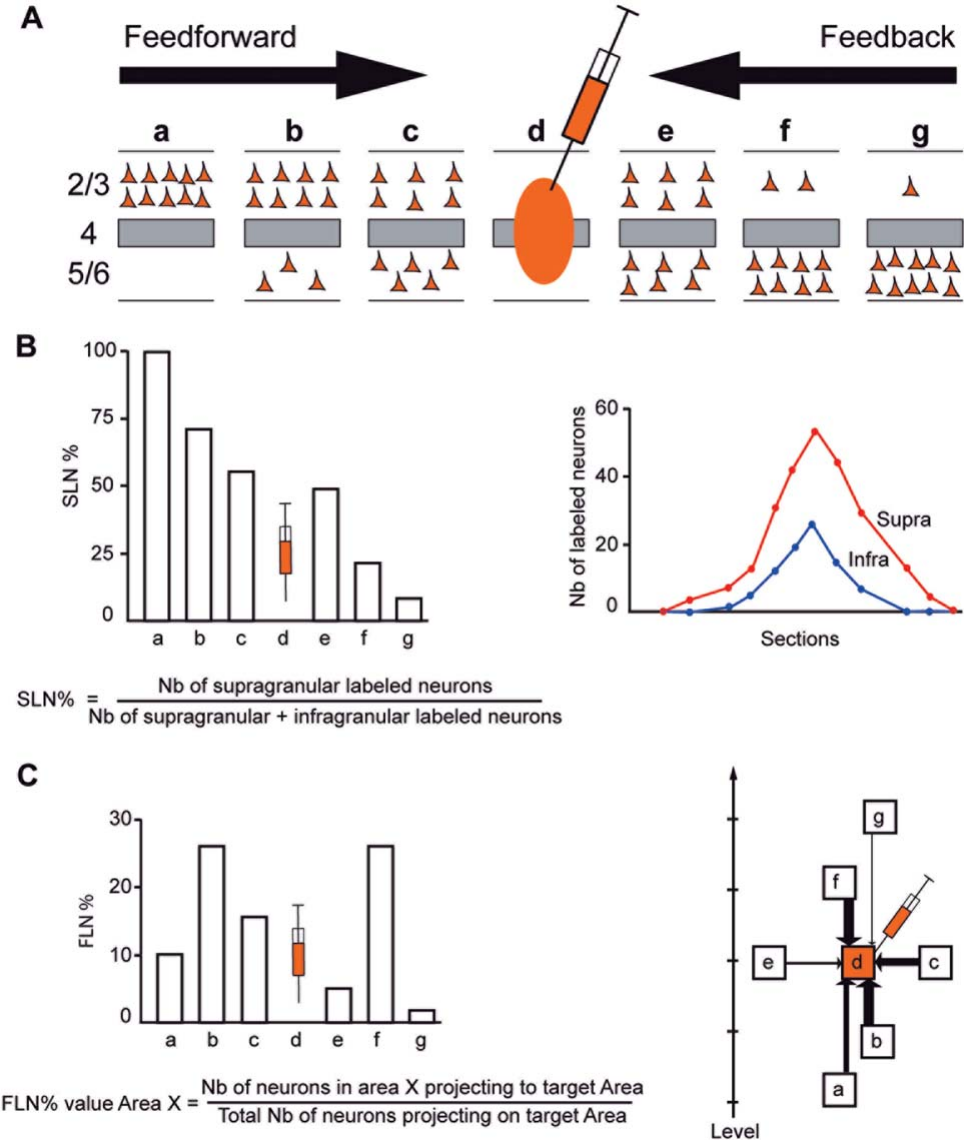
Quantitative parameters characterizing the hierarchy. A: The laminar distribution of parent neurons in each pathway, referred to as SLN (fraction of supragranular neurons) is determined by high-frequency sampling and quantitative analysis of labeling. Supra- and infragranular layer neurons contribute to both FB and FF pathways, and their relative proportion is characteristic for each type of pathway. For a given injection there is a gradient of SLN of the labeled areas, between purely FF (SLN = 100%, all the parent neurons are in the supragranular layers) to purely FB (SLN = 0%, all the parent neurons in the infragranular layers) and a spectrum of intermediate proportions. B: All labeled areas can then be ordered by decreasing SLN values and this order is consistent with hierarchical order according to Felleman and Van Essen (1991). SLN is thus used as an indicator of hierarchical distance between areas from the same injection (Barone et al., 2000; Vezoli et al., 2004). C: FLN (fraction of labeled neurons) indicates the relative strength of each pathway (in number of labeled neurons) compared to the total number of neurons that are labeled in the cortical hemisphere after the injection. It requires counting labeled neurons from sections spanning the whole brain, but gives insight into the weight of connections. Vezoli et al. (2004) showed that short-distance connections have high FLN values, whereas the strength of connection decreases as physical distance between source and target areas increases.

Analysis of FF/FB relations made it possible for Felleman and Van Essen (FVE) to establish a hierarchical ordering of areas, which provided important insight into cortical structure and function (Felleman and Van Essen, 1991). However, a quantitative analysis of the database used by FVE, while confirming a strong hierarchical order, showed that, due to the absence of a distance measure, the FVE model of the visual cortex is indeterminate, with 150,000 equally plausible solutions (Hilgetag et al., 1996, 2000). One possible method of constraining a model of the cortical hierarchy is to use quantitative data on connectivity, which has been shown to provide a measure of hierarchical distance (Barone et al., 2000).

The structural regularities underlying the FVE hierarchy are thought to have a physiological underpinning. Many workers in the field subscribe to the notion that FF signals generate receptive field properties, while FB streams have a modulatory role (Hupé et al., 1998; Ekstrom et al., 2008). The possibility that the interareal pathways are more complicated than this is suggested by reports that physiological activity thought to be characteristic of higher areas can also be found in early visual cortices (Moran and Desimone, 1985; Motter, 1993; Miyashita, 1995; Ishai and Sagi, 1995; Watanabe and Iwai, 1996; Cornette et al., 1998; Lamme et al., 1998; Somers et al., 1999; Super et al., 2001; Lee et al., 2002; Roelfsema et al., 2004). These findings alone suggest that there is no operationally simple definition of higher and lower areas; current theories of visual perception therefore emphasize the complex interactions between different levels of the hierarchy (Cauller, 1995; Pascual-Leone and Walsh, 2001; Tong, 2003; Juan and Walsh, 2003). For instance, it has been suggested that activation of the FF pathways gives rise to rapid automatic characterization with little perceptual detail, the latter being supplied by reiterative engagement of FB pathways (Pascual-Leone and Walsh, 2001; Hochstein and Ahissar, 2002; Juan and Walsh, 2003; Lamme, 2003; Tong, 2003; Jehee et al., 2007).

The debate on the respective roles of FF and FB pathways is clearly still open. Furthermore, the nature of the interaction between higher and lower areas could be partly shaped by the structural aspects of these pathways. FF and FB pathways in the visual system are reported to show strong asymmetry in structural features. Two general claims have been made. First, it is argued that FF connections are topologically organized, in contrast to a more diffusely ordered FB connections, both in terms of the spatial extent of parent neurons and terminals and of the frequency of axonal bifurcation (Rockland and Pandya, 1979; Maunsell and van Essen, 1983; Bullier et al., 1984; Kennedy and Bullier, 1985; Ungerleider and Desimone, 1986; Bullier and Kennedy, 1987; Zeki and Shipp, 1988, Krubitzer and Kaas, 1989, 1990; Shipp and Zeki, 1989; Rockland and Virga, 1989; Henry et al., 1991; Shipp and Grant, 1991; Salin et al., 1992; Rockland and Van Hoesen, 1994). Second, it is argued that FB pathways are more numerous and cross more hierarchical levels than do FF pathways (Zeki, 1978; Doty, 1983; Kennedy and Bullier, 1985; Yukie and Iwai, 1985; Perkel et al., 1986; Iwai and Yukie, 1988; Webster et al., 1991; Nakamura et al., 1993).

Because the nature of the FF and FB pathways is at the heart of our understanding of cortical hierarchy, their organizational principles will have important consequences for any general theory of cortical function. Hierarchical processing is central to theories of cortical function in which perception is considered an inference derived from the interaction of incoming sensory information with stored generative representations (Mumford, 1992). Generative models argue that structural cortical hierarchy ensures reiterative interactions between prediction errors ascending the hierarchy and predictions descending the hierarchy, and the functional asymmetries of the FF and FB are thought to reflect these two processes (Friston, 2003; Markov and Kennedy, 2013). One development of the generative model insists on the equivalence and segregation of the streams, forming an interareal counterstream that converges at the level of the area in order to interact with the local cortical processing of the cortex (Ullman, 1995). In Ullman’s proposition, an FF was located in the supragranular layers and an FB stream in the infragranular layers. The counterstream theory makes a number of testable predictions, the most significant being that FF and FB pathways are highly segregated. Previous studies have shown that FF and FB connections are not restricted respectively to the supragranular or infragranular layer; but instead both streams involve varying proportions of cells of both of these compartments (Barone et al., 2000). According to the counterstream theory, the pyramidal neurons that project to lower cortical areas should not possess axon collaterals projecting to higher cortical areas, as this would compromise the segregation of the two counterstreams. Because FB projections are known to send substantial numbers of collaterals to more than one target area, this is still an open issue that needs to be tested (Rockland, 2004, 2013).

Elsewhere we show that weight and distance relations obtained from retrograde tracing experiments predict numerous properties of the cortical network, including the specificity of the long-range connections between lobes, global and local efficiency, and the optimal placement of areas (Ercsey-Ravasz et al., 2013; Markov et al., 2013). Here we implement a similar approach involving weight and distance focusing on the structural features of the FF and FB pathways in the visual cortex. First, the percentage of supragranular labeled neurons (SLN: see Materials and Methods), a quantitative measure of the laminar distribution of parent neurons of cortical projections was used to model the hierarchal organization of early visual areas. Second, SLN was shown to reflect a combinatorial distance rule of supra- and infragranular projecting neurons. Third, the relative strength and number of FF and FB pathways were determined. Fourth, FF and FB pathway topography was examined. And fifth, the segregation of the two pathways was estimated.

## MATERIALS AND METHODS

All the procedures used in the study followed the national and European regulations concerning animal experiments (EC guidelines 86/609/EC) and were approved by the authorized national and veterinary agencies.

### Anesthesia and surgery

Twenty-six macaque monkeys (*Macaca fascicularis* and *Macaca mulatta,* Table 1) were premedicated for surgery with atropine (1.25 mg, i.m.) and dexamethasone (4 mg, i.m.). The animals were then anesthetized with ketamine hydrochloride (20 mg/kg, i.m.) and chlorpromazine (2 mg/kg, i.m.). Heart rate was monitored and artificial respiration was adjusted to maintain end-tidal CO_2_ at 4.5–6%. Rectal temperature was maintained at 37°C. A surgical plane of anesthesia was maintained with 1–2% halothane in N_2_O and O_2_ (70:30).

**Table 1:** Animal Cases and Procedures

Animal	Hemisphere/tracers	Injection site	Plane of section	Paper (P) /Mercator (M)	Frequency of examination of section	Sex	Age
M81	LH / DY	V1	H	M	1/2	F	Adult
M85	LH / FB	V1	H	M	1/2	F	Adult
M85	RH/FB+DY	V1	H	M	1/2	F	Adult
M88	RH / FB	V1	H	M	1/2	F	Adult
M121	RH / DY	V1	C	M	1/2	F	Adult
M71	RH/FB+DY	V1	P	P	1/8	F	Adult
M37	LH/FB+DY	V1	H	P	1/2	F	Adult
BB75	RH/FB+DY	V1	P	P	1/4	M	1.5 months
M73	LH / DY	V1	P	P/M	1/4	M	Adult
M101^1^	LH / DY	V2	C	M	1/2	M	Adult
M101^1^	RH / FB	V2	C	M	1/2	M	Adult
M103	LH / DY	V2	C	M	1/2	M	Adult
M121	RH / FB	V4	C	M	1/2	F	Adult
M123	LH / DY	V4	C	M	1/2	M	Adult
M72	LH/ FB+DY	V4	H	P/M	1/4	F	Adult
BB119	LH/ FB+DY	V4	H	P	1/4	M	2 months
BB187	LH/ FB+DY	V4	H	P	1/4	M	2 months
M73	LH/FB	V4	P	P/M	1/4	M	Adult
M119	LH / FB	TEO	C	M	1/2	F	Adult
BB272	LH / DY	8m	C	M	1/2	F	6 months
BB135	LH / DY	7A	H	P/M	1/4	F	12 months
M89	LH / DY	DP	H	M	1/2	F	Adult
M90	RH / FB	STPc	H	M	1/4	F	Adult
M128	LH / FB	TEpd	C	M	1/2	F	Adult
BB272	RH/ FB	8L	C	M	1/2	F	6 months
M133	LH / DY	MT	C	M	1/2	F	Adult
M132	LH	Used to build the atlas	C	M	1/2	F	Adult

Animal	Hemisphere/tracers		Injection site		Sex	Age (months)	
BB270	RH/DY		V1		M	0.5	
BB271	LH/DY		V1		F	0.75	
BB273	LH/DY		V1/V4		M	2	
BB273	RH/DY		V1		M	2	
BB274	LH/DY		V4		M	1	

P corresponds to charts of neurons stored on paper, and M to charts made and stored with Mercator technology (see Materials and Methods).

1 Rhesus macaque.

### Injection of retrograde tracers

Injections were made using an image-guided stereotaxic system (Brainsight Frameless, Rogue Research, Montreal, Canada). The target area was identified on the monkey’s magnetic resonance imaging (MRI) using sulcal landmarks in a 3D reconstruction of the monkey brain and a coronal, parasagittal, or horizontal plane (Frey et al., 2004). The Brainsight system monitors injection position online and to within a few millimeters range. Injections of the fluorescent Fast blue and Diamidino yellow tracers (0.2–0.3 μl) spanning the full depth of the cortex were made into V1, V2, V4, TEO, TEpd, MT, 7a, STPc, DP, 8m, and 8L. Injection sites can be viewed in Markov et al. (2013).

The spatial extent of labeling and the percentages of double-labeled neurons in supragranular vs. infragranular layers (in V2, V3, MT, TEO, and TE) were computed after paired parallel longitudinal injection of 3–5 μl of the two tracers in V1 in one brain and in V4 in another brain. These paired injections, 2–3 mm apart, were used to quantify the divergence of terminal arbors and the degree of scatter in projection topology, and were made at a shallow angle to the cortical surface spanning the entire thickness of the cortical sheet. The tracer was injected while the Hamilton microsyringe was withdrawn from the cortex so as to form parallel longitudinal injection sites restricted to the cortical gray matter.

In order to quantify the frequency of single neurons sending projections to both V1 and V4, simultaneous injections were made in these two areas. In one animal, massive injections were made by multiple injection of Diamidino yellow in the opercular part of V1 and, in the same hemisphere, Fast blue was massively injected in V4 between the lunate sulcus and the superior temporal sulcus. Both sets of injections involved corresponding regions representing the lower part of the central visual field (Gattass et al., 1987, 1988).

Following a 10–13 day survival period, to allow retrograde transport of the tracers, the animals were deeply anesthetized and perfused through the heart with 2.7% saline, followed by 4–8% paraformaldehyde, 0.05% glutaraldehyde in 0.1 M phosphate buffer (PB) (pH 7.4), and 10–30% sucrose in PB. The brains were then blocked in the coronal, sagittal, or horizontal plane, and 40-μm-thick sections were cut on a freezing microtome. One in three sections was immediately mounted from saline solution onto 3% gelatin-coated slides. Selected sections at regular intervals from those not used for counting were reacted for cytochrome oxidase, acetylcholinesterase (AChE) activity (Barone et al., 2000), and SMI-32 (Hof et al., 1996). Sections were observed with a Leitz or Leica DMRE fluorescence microscope equipped with a D-filter set (355–425 nm). A computer-assisted program (ExploraNova) was used with a motorized microscope stage so as to trace out sections electronically and record neuron positions with high precision (±10 μm).

### Examination of material

The sections were left without coverslips and were observed with oil-immersion objectives under UV light with a fluorescent microscope equipped with a D-filter set (355–425 nm). The characteristics of neurons labeled with Fast blue and Diamidino yellow have been described elsewhere (Keizer et al., 1983): neurons labeled by Fast blue exhibit a blue coloration in their cytoplasm while those labeled by Diamidino yellow exhibit a yellow nucleus. After plotting, sections were counterstained for Nissl substance and backprojected onto the charts of labeled neurons so as to trace cytoarchitectonic areal and laminar borders.

Accurate estimates were made of the numbers of neurons per area with respect to the total number of neurons encountered in one cortical hemisphere by plotting 1/3 sections throughout the brain (Vezoli et al., 2004). The fraction was expressed as the FLN (fraction of labeled neurons) and the percentage of SLN (Fig. 1) (Barone et al., 2000).

SLN values and distances are listed in Table 2. FLN values are published in Markov et al. (2013). Tracer injection leads to dense labeling of extensive regions of the cortex. The full set of source areas projecting to each of our injected target areas is reported elsewhere (Markov et al., 2012). The present study used the totality of labeled neurons to estimate the FLN values but restricted the list of source areas considered to those that have their analog in the FVE hierarchical model. The source areas reported in this study include V1, V2, V3, V3A, V4, V4t, 7A, 7B, LIP, STPr, STPi, STPc, FST, MST, MT, TEpd, TEpv, TEad, TEav, TEa/ma, TEa/mp, 8L, 8m, TEO, TEOm, DP, V6, V6A, VIP, PIP, TF, TH, MIP, 7m, 9/46d, 9/46v, 46v, 46d, perirhinal (Peri), and entorhinal (Ento). A full description of atlases can be found at http://www.core-nets.org. Multiple criteria were used to allocate labeled neurons to particular extrastriate areas, as described elsewhere (Hof and Morrison, 1995; Stepniewska and Kaas, 1996; Kaas and Hackett, 1998; Barone et al., 2000; Falchier et al., 2002; Clavagnier et al., 2004; Markov et al., 2011, 2012).

**Table 2:** SLN and Distance Values

To	From	SLN (%)	Dist (mm)
7A	V1	0.00	25.5
7A	V2	50.00	24.5
7A	V3A	50.00	15.4
7A	V4	80.18	22.8
7A	7B	5.77	12.8
7A	LIP	58.11	6.3
7A	VIP	66.67	10.3
7A	MIP	0.00	19.4
7A	PIP	40.00	9.2
7A	DP	23.15	11.4
7A	V6	14.29	18.2
7A	V6A	44.20	24.6
7A	5	38.74	22.5
7A	7m	42.07	18.1
7A	STPr	64.13	29
7A	STPi	52.35	21
7A	STPc	65.88	10.2
7A	TPt	54.31	6.3
7A	PGa	32.44	21.7
7A	IPa	12.50	23.6
7A	FST	45.55	18.1
7A	MST	61.21	6.8
7A	MT	100.00	18.8
7A	TEO	62.39	24.6
7A	TEOm	78.17	27.4
7A	PERIRHINAL	20.98	37.2
7A	TEav	0.00	32.1
7A	TEpd	6.54	28.3
7A	TEpv	36.24	27.2
7A	TEa/ma	0.00	34.8
7A	TEa/mp	46.28	29.1
7A	ENTORHINAL	1.88	37
7A	TH/TF	22.34	27.9
7A	TEMPORAL_POLE	0.00	34.5
7A	MB	71.43	23.8
7A	LB	0.00	20.8
7A	INSULA	37.59	22.8
7A	2	0.00	17.9
7A	23	41.29	16
7A	24a	23.33	26.8
7A	24b	34.55	26.7
7A	29/30	54.86	16.6
7A	31	41.30	15.9
7A	F1	100.00	23
7A	F2	72.59	27.1
7A	F7	23.53	35.2
7A	F3	56.36	28.2
7A	F5	96.55	29.2
7A	9	22.22	40
7A	46d	67.67	36.4
7A	46v	0.00	37.2
7A	9/46d	44.27	35.5
7A	9/46v	47.37	34.8
7A	8B	44.05	35.4
7A	8L	49.02	30.4
7A	8m	30.51	30.6
7A	45B	40.00	32.5
7A	45A	71.81	33.6
7A	12	0.00	36.4
7A	13	0.00	33.9
8L	V1	75.00	45.6
8L	V2	92.86	40.2
8L	V3	92.52	42
8L	V3A	81.25	40
8L	V4	95.69	40.2
8L	V4t	68.97	40.6
8L	7op	54.55	26.3
8L	7A	25.58	30.4
8L	7B	66.67	22.6
8L	LIP	50.44	33.4
8L	VIP	30.54	28.3
8L	MIP	37.50	34.5
8L	PIP	75.60	40
8L	AIP	40.00	21.3
8L	DP	77.97	37.5
8L	V6A	4.76	42
8L	5	16.67	31.3
8L	7m	8.33	32.3
8L	STPr	10.91	21.4
8L	STPi	15.13	23.9
8L	STPc	32.48	28.8
8L	TPt	40.48	33.3
8L	PGa	40.67	23.3
8L	IPa	30.77	26
8L	FST	56.39	31.9
8L	MST	42.29	38.5
8L	MT	48.78	31.8
8L	TEO	83.33	32.3
8L	TEOm	88.33	38.5
8L	PERIRHINAL	80.00	24.3
8L	TEad	100.00	29.3
8L	TEav	38.46	24.3
8L	TEpd	53.33	31
8L	TEpv	61.29	29.5
8L	TEa/ma	9.09	28
8L	TEa/mp	80.00	30.7
8L	ENTORHINAL	25.00	22.9
8L	TH/TF	54.84	35.7
8L	SUBICULUM	0.00	34.2
8L	TEMPORAL_POLE	20.00	22.3
8L	CORE	10.94	27.8
8L	MB	10.64	24.5
8L	LB	31.58	29.9
8L	PBr	14.63	26.1
8L	PBc	42.86	33
8L	Parainsula	15.63	18.3
8L	INSULA	10.20	21.3
8L	Gu	4.65	16
8L	SII	17.80	18.3
8L	2	45.00	21.8
8L	3	58.33	23
8L	23	54.55	28.7
8L	24a	56.23	14.3
8L	24b	33.19	14.3
8L	24c	39.08	11.2
8L	24d	43.69	16.3
8L	29/30	50.00	26.4
8L	31	28.57	33.7
8L	32	50.00	17.9
8L	F1	19.57	20.7
8L	F2	19.57	18.1
8L	F7	25.80	13.5
8L	F3	9.09	17
8L	F6	35.09	14.5
8L	F4	22.41	16.3
8L	F5	31.85	14.4
8L	ProM	7.02	14.7
8L	10	7.69	23.6
8L	9	17.46	16.7
8L	46d	27.27	16.4
8L	46v	16.71	15.2
8L	9/46d	21.13	12.4
8L	9/46v	29.66	9.3
8L	8B	25.16	16.1
8L	8m	49.87	6.1
8L	8r	59.99	3.8
8L	45B	38.15	8.3
8L	45A	30.00	10.1
8L	44	36.59	8.7
8L	OPRO	8.58	12.7
8L	OPAI	15.79	15.6
8L	11	26.42	15.9
8L	14	66.67	17.5
8L	25	40.00	16.5
8L	12	29.18	16.2
8L	13	7.80	13.5
8L	PIRIFORM	88.89	14.7
8m	V2	20.00	40.3
8m	V3	25.00	42
8m	V3A	0.00	38.9
8m	V4	100.00	38.8
8m	V4t	58.33	39.4
8m	7op	46.81	25
8m	7A	30.30	30.6
8m	7B	66.67	22.5
8m	LIP	24.61	32.5
8m	VIP	34.48	27.5
8m	MIP	100.00	33.3
8m	PIP	0.00	38.8
8m	AIP	41.33	22
8m	DP	70.59	41.3
8m	V6A	43.75	40.8
8m	5	57.14	30.8
8m	7m	51.11	31.4
8m	STPr	5.08	20.5
8m	STPi	20.90	23.6
8m	STPc	26.96	28.9
8m	TPt	14.29	30.3
8m	PGa	29.71	21.7
8m	IPa	12.50	24.4
8m	FST	27.00	30.3
8m	MST	44.38	32.5
8m	MT	59.65	31.7
8m	TEOm	50.00	37.1
8m	PERIRHINAL	0.00	24.2
8m	TEpd	33.33	31.5
8m	TEpv	0.00	28.6
8m	TEa/ma	66.67	27.4
8m	TEa/mp	15.38	30.2
8m	ENTORHINAL	0.00	22.3
8m	TH/TF	0.00	34.2
8m	TEMPORAL_POLE	18.18	23.8
8m	CORE	22.81	26.2
8m	MB	16.31	23.1
8m	LB	33.68	28.4
8m	PBr	18.56	26.2
8m	PBc	25.06	31.9
8m	Parainsula	10.00	18.3
8m	INSULA	28.39	18.5
8m	Gu	36.36	17.5
8m	SII	37.10	16.7
8m	2	0.00	21.2
8m	3	50.00	22
8m	23	27.27	28.1
8m	24a	20.69	13.9
8m	24b	12.05	13.9
8m	24c	20.51	9
8m	24d	36.67	15.8
8m	29/30	0.00	25.8
8m	31	33.33	33.1
8m	32	8.33	16.9
8m	F1	29.41	20.2
8m	F2	20.30	13.9
8m	F7	28.24	13
8m	F3	26.32	17.2
8m	F6	12.50	15.3
8m	F4	30.50	15.6
8m	F5	35.37	14.1
8m	ProM	39.58	16.5
8m	10	1.16	22.8
8m	9	22.02	17.8
8m	46d	30.56	15.4
8m	46v	40.15	20
8m	9/46d	36.96	12.5
8m	9/46v	47.34	13.1
8m	8B	15.73	12.1
8m	8L	57.70	6.1
8m	8r	48.79	7.3
8m	45B	55.55	11.7
8m	45A	53.60	13.7
8m	44	39.37	9.6
8m	OPRO	14.29	14.9
8m	OPAI	0.00	14.5
8m	11	13.33	20.4
8m	14	37.50	21.1
8m	12	28.79	17
8m	13	21.33	14.3
MT	V1	89.05	12.5
MT	V2	94.17	13.6
MT	V3	89.64	11
MT	V3A	86.33	8
MT	V4	61.53	9.4
MT	V4t	47.07	10.9
MT	Pro.St.	33.33	13.2
MT	7A	66.12	18.8
MT	LIP	52.91	13.2
MT	VIP	51.39	13.9
MT	MIP	0.00	24.6
MT	PIP	61.48	14.5
MT	DP	83.77	15.6
MT	V6A	100.00	17.1
MT	STPr	1.23	26.6
MT	STPi	2.74	19.8
MT	STPc	5.02	19
MT	TPt	44.44	15.6
MT	PGa	3.93	14.5
MT	IPa	5.59	16.7
MT	FST	28.59	8.9
MT	MST	18.69	16.2
MT	TEO	33.27	9.7
MT	TEOm	36.21	10.8
MT	PERIRHINAL	0.34	27.3
MT	TEad	1.69	24.5
MT	TEav	0.51	24.9
MT	TEpd	20.79	16.6
MT	TEpv	23.47	14.8
MT	TEa/ma	5.78	25.5
MT	TEa/mp	32.44	17.9
MT	TH/TF	16.51	14.3
MT	SUBICULUM	NA	18.7
MT	TEMPORAL_POLE	0.00	31
MT	MB	7.41	18.3
MT	LB	100.00	21.8
MT	PBr	0.00	24.3
MT	INSULA	6.67	18.7
MT	SII	0.00	23.7
MT	1	100.00	23
MT	2	0.00	20.5
MT	23	2.63	17
MT	24a	0.00	28.5
MT	24b	0.00	28.7
MT	29/30	16.67	17.2
MT	32	0.00	36.6
MT	F1	0.00	24.5
MT	F2	50.00	29.1
MT	F4	14.29	25.9
MT	F5	0.00	31.3
MT	ProM	0.00	33.7
MT	9/46d	100.00	36.4
MT	9/46v	84.62	35.6
MT	8L	67.28	31.8
MT	8m	40.76	31.7
MT	8r	70.37	33.4
MT	45B	45.24	33.7
MT	45A	0.00	35
MT	OPRO	100.00	30
STPc	V2	21.43	28.2
STPc	V3A	100.00	20.8
STPc	V4	52.04	25.7
STPc	Pro.St.	0.00	16.1
STPc	7op	68.97	14.8
STPc	7A	44.12	10.2
STPc	7B	37.50	18.3
STPc	LIP	67.10	10.4
STPc	VIP	0.00	13.3
STPc	PIP	70.52	16.1
STPc	DP	78.57	15.2
STPc	V6A	0.00	21.5
STPc	5	100.00	24.5
STPc	7m	0.00	19.1
STPc	STPr	15.60	22.2
STPc	STPi	61.14	12.7
STPc	TPt	60.74	5.9
STPc	PGa	46.61	13.9
STPc	IPa	33.83	20.9
STPc	FST	46.82	12.2
STPc	MST	58.22	6
STPc	MT	42.31	19
STPc	TEO	50.00	24.8
STPc	TEOm	15.91	24.1
STPc	PERIRHINAL	4.26	25.5
STPc	TEad	1.85	26.3
STPc	TEav	3.57	24.6
STPc	TEpd	28.07	20.7
STPc	TEpv	10.89	19.4
STPc	TEa/ma	13.51	27.2
STPc	TEa/mp	29.63	20.1
STPc	ENTORHINAL	0.00	24.6
STPc	TH/TF	4.55	21.4
STPc	SUBICULUM	NA	24
STPc	TEMPORAL_POLE	3.31	28.9
STPc	CORE	77.91	13.5
STPc	MB	64.32	13.7
STPc	LB	66.04	14.2
STPc	PBr	21.09	21.3
STPc	PBc	53.22	10.6
STPc	Parainsula	12.90	20.2
STPc	INSULA	66.62	20.4
STPc	2	20.00	22
STPc	23	19.37	17.9
STPc	24b	42.42	26.7
STPc	24c	0.00	32.5
STPc	29/30	0.00	19
STPc	31	10.32	17.2
STPc	F7	27.45	24.1
STPc	F6	100.00	35.4
STPc	F5	60.27	28.9
STPc	10	41.18	41.8
STPc	9	50.00	38.4
STPc	46d	63.96	35.3
STPc	46v	58.33	36.3
STPc	9/46d	31.48	34.4
STPc	8B	50.00	34.5
STPc	8L	80.56	28.8
STPc	8m	55.38	28.9
STPc	8r	51.43	29.7
STPc	45B	46.30	30.3
STPc	45A	0.00	31.2
STPc	12	32.99	31.6
STPc	13	18.57	31.4
TEO	V2	93.70	12.2
TEO	V3	84.25	11.3
TEO	V3A	33.77	15.3
TEO	V4	66.41	9.8
TEO	V4t	52.02	9.4
TEO	7A	0.00	24.6
TEO	7B	100.00	28.7
TEO	LIP	30.84	20.8
TEO	MIP	0.00	27.4
TEO	PIP	48.31	24.8
TEO	DP	26.37	21.3
TEO	5	100.00	30.4
TEO	7m	37.50	25.6
TEO	STPr	7.32	25.5
TEO	STPi	0.00	21.5
TEO	STPc	0.00	24.8
TEO	PGa	3.95	16.2
TEO	IPa	11.71	15.5
TEO	FST	37.19	7.2
TEO	MST	0.00	23.1
TEO	MT	42.42	9.7
TEO	TEOm	48.28	7
TEO	PERIRHINAL	3.77	22.7
TEO	TEad	23.38	19.2
TEO	TEav	30.68	21.2
TEO	TEpd	34.97	10.7
TEO	TEpv	30.44	10.8
TEO	TEa/ma	47.40	19.6
TEO	TEa/mp	30.77	13.1
TEO	ENTORHINAL	100.00	24.2
TEO	TH/TF	2.37	14.2
TEO	TEMPORAL_POLE	18.37	30.6
TEO	MB	0.00	18.3
TEO	LB	100.00	23.3
TEO	PBr	25.00	28.5
TEO	PBc	100.00	22.3
TEO	Parainsula	71.43	29.6
TEO	INSULA	30.00	20.3
TEO	1	0.00	26.9
TEO	2	0.00	25.9
TEO	3	21.05	24
TEO	23	100.00	23.4
TEO	24a	0.00	20.8
TEO	24b	0.00	31.2
TEO	24d	33.33	31.6
TEO	F2	100.00	33.6
TEO	F7	85.71	37.9
TEO	F5	83.33	31.4
TEO	ProM	100.00	38.8
TEO	46d	50.00	40.1
TEO	46v	50.00	40
TEO	9/46d	100.00	38.9
TEO	9/46v	67.21	36.5
TEO	8B	0.00	38
TEO	8L	74.92	32.3
TEO	8r	79.76	33.8
TEO	45B	50.77	33.7
TEO	45A	66.67	34.6
TEO	44	50.00	28.4
TEO	11	0.00	41.2
TEO	12	37.84	39.6
TEpd	V2	97.14	19.8
TEpd	V3	92.11	21.9
TEpd	V3A	91.43	26.8
TEpd	V4	95.30	16.5
TEpd	V4t	91.30	16.6
TEpd	Pro.St.	10.00	24.1
TEpd	7A	50.37	28.3
TEpd	LIP	72.16	25.2
TEpd	PIP	86.36	29.8
TEpd	DP	68.75	28.8
TEpd	STPr	20.47	18.2
TEpd	STPi	20.00	16.6
TEpd	STPc	0.00	20.7
TEpd	PGa	11.30	14.7
TEpd	IPa	17.89	9.3
TEpd	FST	22.54	12.5
TEpd	MST	100.00	23.1
TEpd	MT	33.33	16.6
TEpd	TEO	67.97	10.7
TEpd	TEOm	70.97	10.6
TEpd	PERIRHINAL	7.55	18.4
TEpd	TEad	51.65	11.2
TEpd	TEav	44.90	14
TEpd	TEpv	28.04	3.6
TEpd	TEa/ma	36.32	12.3
TEpd	TEa/mp	46.51	4.9
TEpd	ENTORHINAL	10.22	19.3
TEpd	TH/TF	3.59	13.4
TEpd	TEMPORAL_POLE	0.86	24.5
TEpd	CORE	0.00	18.8
TEpd	MB	0.00	16.1
TEpd	LB	0.00	20.4
TEpd	PBr	0.00	19.9
TEpd	Parainsula	0.00	21.8
TEpd	INSULA	0.00	14.9
TEpd	SII	22.34	26
TEpd	3	100.00	23.2
TEpd	23	0.00	26.4
TEpd	24a	20.37	30.2
TEpd	24b	14.29	30.2
TEpd	F6	66.67	37.8
TEpd	F5	0.00	28.7
TEpd	46d	0.00	38
TEpd	46v	42.94	37.4
TEpd	9/46v	48.59	33.2
TEpd	8L	72.50	31
TEpd	8m	20.00	31.5
TEpd	8r	59.66	32
TEpd	45B	67.54	30.5
TEpd	45A	71.28	31.5
TEpd	44	27.27	26.5
TEpd	OPRO	12.50	25.7
TEpd	OPAI	100.00	26.2
TEpd	11	14.29	37.8
TEpd	12	50.48	35.8
TEpd	13	56.62	33.6
TEpd	PIRIFORM	37.50	23.9
V1	V2	42.08	9.3
V1	V3	6.70	4.5
V1	V3A	0.44	10.5
V1	V4	29.65	14.8
V1	V4t	2.26	13.9
V1	7op	33.33	29
V1	LIP	0.91	22.7
V1	PIP	0.00	20.1
V1	DP	0.40	16.5
V1	STPi	8.17	28.3
V1	STPc	3.62	29.5
V1	TPt	50.00	28
V1	PGa	0.38	24
V1	IPa	1.75	26.1
V1	FST	2.09	16.7
V1	MST	0.88	23.2
V1	MT	17.32	12.5
V1	TEO	9.60	14.9
V1	TEOm	0.99	19.2
V1	PERIRHINAL	0.72	40
V1	TEad	0.59	33.2
V1	TEav	0.00	36.3
V1	TEpd	2.05	25.5
V1	TEpv	0.19	23.8
V1	TEa/ma	2.92	35.5
V1	TEa/mp	2.13	27.2
V1	TH/TF	0.59	24.4
V1	CORE	0.00	30.3
V1	MB	0.00	27.2
V1	LB	6.25	32.7
V1	PBc	27.76	33.3
V1	8L	10.42	45.6
V1	STPr	4.76	36.8
V1	8r	0.00	46.4
V2	V1	73.60	9.3
V2	V3	32.14	10.2
V2	V3A	2.75	14.9
V2	V4	25.45	9.4
V2	V4t	23.76	10.7
V2	LIP	4.92	21.4
V2	VIP	0.76	21
V2	PIP	0.63	19.9
V2	DP	7.46	21.6
V2	V6A	38.10	22.7
V2	STPi	7.41	27
V2	STPc	0.00	28.2
V2	TPt	0.00	25.2
V2	PGa	2.33	24.6
V2	IPa	37.04	24.2
V2	FST	7.05	16.2
V2	MST	1.88	23.4
V2	MT	26.86	13.6
V2	TEO	9.10	12.2
V2	TEOm	5.14	13.2
V2	PERIRHINAL	3.50	30
V2	TEad	1.67	27
V2	TEav	1.42	29.2
V2	TEpd	3.24	19.8
V2	TEpv	1.92	19.3
V2	TEa/ma	0.00	28.1
V2	TEa/mp	5.26	22.8
V2	TH/TF	1.00	19
V2	MB	0.00	26
V2	8L	24.25	40.2
V2	8m	0.00	40.3
V2	V6	0.00	20.8
V2	STPr	40.00	34.3
V2	PBc	0.00	30.2
V4	V1	98.17	14.8
V4	V2	92.60	9.4
V4	V3	65.94	12.9
V4	V3A	0.00	12.4
V4	V4t	43.93	3.9
V4	7A	4.35	22.8
V4	LIP	21.54	21.4
V4	PIP	14.88	14.9
V4	DP	0.00	15
V4	STPr	7.41	34.6
V4	STPi	0.00	26.9
V4	STPc	0.00	25.7
V4	PGa	2.50	24.4
V4	IPa	6.21	25.3
V4	FST	16.59	15.5
V4	MST	4.35	23.6
V4	MT	46.08	9.4
V4	TEO	43.04	9.8
V4	TEOm	24.84	9.1
V4	PERIRHINAL	0.04	29.7
V4	TEad	1.30	24.2
V4	TEav	2.44	26.4
V4	TEpd	27.47	16.5
V4	TEpv	3.90	14.8
V4	TEa/ma	3.56	24.5
V4	TEa/mp	15.52	17.3
V4	ENTORHINAL	0.00	29.3
V4	TH/TF	1.21	19.4
V4	LB	100.00	29.6
V4	INSULA	48.33	29
V4	9/46v	0.00	43.8
V4	8L	60.42	39.5
V4	45B	25.00	41.5
V4	9/46d	0.00	45.9
V4	8r	47.37	41.1
DP	V1	0.00	16.5
DP	V2	91.50	21.6
DP	V3	92.43	12.9
DP	V3A	71.90	12
DP	V4	50.14	15
DP	V4t	21.92	14.1
DP	Pro.St.	20.90	17.2
DP	7A	34.46	11.4
DP	LIP	36.81	11.9
DP	VIP	6.09	14.9
DP	MIP	25.18	20.6
DP	PIP	43.73	5.9
DP	V6	67.91	19.4
DP	V6A	58.22	22.9
DP	7m	0.00	21.1
DP	STPr	100.00	33.9
DP	STPi	0.00	26.6
DP	STPc	0.00	15.2
DP	TPt	8.33	13.4
DP	PGa	9.29	23.3
DP	IPa	23.47	29.3
DP	FST	26.64	20.2
DP	MST	30.21	13.8
DP	MT	48.12	15.6
DP	TEO	15.29	21.3
DP	TEOm	34.95	20.7
DP	PERIRHINAL	20.00	34.4
DP	TEad	0.00	36.2
DP	TEav	0.00	34.9
DP	TEpd	16.67	28.8
DP	TEpv	43.50	31.2
DP	TEa/ma	8.60	37.1
DP	TEa/mp	3.70	32.1
DP	ENTORHINAL	5.17	32.4
DP	TH/TF	20.49	33.1
DP	SUBICULUM	NA	34.5
DP	PBc	0.00	25.5
DP	INSULA	100.00	25.2
DP	23	18.98	18.6
DP	24b	34.21	33.9
DP	24c	0.00	35.7
DP	29/30	63.00	19.7
DP	31	32.73	17.2
DP	F2	33.07	32.9
DP	F7	8.59	38.6
DP	F4	0.00	31.8
DP	F5	0.00	34.3
DP	9	100.00	44.5
DP	46d	48.95	41.5
DP	46v	9.09	45.1
DP	9/46d	36.55	38.4
DP	8B	36.04	40.2
DP	8m	51.65	41.3
DP	8r	42.34	36.8
DP	45B	78.82	37.5
DP	45A	75.00	38.4
DP	12	0.00	45.8

**Figure 2:**
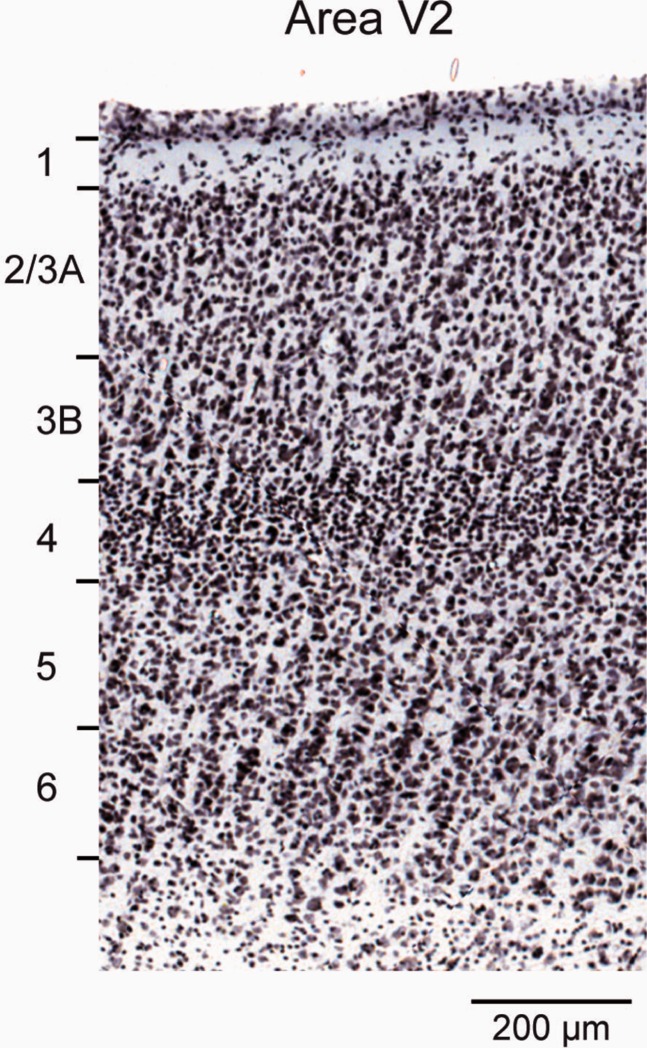
Laminar limits of area V2.

A central aspect of the present study is the laminar location of parent neurons of FF and FB pathways. In area V1, labeled neurons in layer 4B were classified as SLNs. In extrastriate cortex two distinct compartments were distinguished in the supragranular layers: layers 2/3A and 3B (Fig. 2).

### Cell morphology experiments

Four juvenile monkeys were used (21–60 postnatal days). Diamidino yellow (2.0%) was injected in two sets of animals in area V1 (three animals) and V4 (two animals). FF and FB neurons were examined in area V2. After a 10–13-day survival period, the monkey was deeply anesthetized and blocks of fresh brain removed.

The 300-μm-thick parasagittal slices were cut with a vibroslicer (Leica VT1000S) in ice-cold slicing solution containing (in mM): choline chloride (116.5), KCl (2.5), CaCl_2_ (0.5), MgCl_2_ (6), NaH_2_PO_4_ (1.25), NaHCO_3_ (25) and glucose (25), continuously bubbled with 95% O_2_-5% CO_2_ (pH = 7.4).

Slices were incubated at 37°C for 1 hour before use in carbogenated artificial cerebrospinal fluid (ACSF) containing (in mM): NaCl (120), KCl (2.5), CaCl_2_ (2.5), MgCl_2_ (1), NaH_2_PO_4_ (1.25), NaHCO_3_ (25), and glucose (25) (pH = 7.4).

Retrogradely labeled cells were filled during whole-cell patch clamp recording (not shown) with biocytin (2 mg/ml) dissolved in the internal solution, which also contained (in mM): K-Gluconate (140), HEPES (10), EGTA (0.2), and MgATP (4), pH 7.2. To ensure complete cell filling by diffusion of the biocytin, the whole cell configuration was maintained for at least 30 minutes.

Slices containing biocytin-filled neurons were fixed overnight in 4% paraformaldehyde in 0.1M PB saline solution (PBS) at 4°C. After washing, slices were incubated for 30 minutes with 0.3% Triton in PBS (PBS-T) and then incubated for 4 hours with Cy3-conjugated streptavidin (1/200) (Invitrogen, La Jolla, CA) in PBS-T. After washing in PBS, sections were counterstained with Bisbenzimide (1 μg/ml) to allow identification of cortical layers.

3D stacks of images were obtained on a Leica confocal microscope (×40 lens). Reconstruction and quantitative analysis used Amira software. Statistical analysis was performed using the Wilcoxon nonparametric test.

### Statistical analysis

All statistical tests were performed in the Open Source software R. (R_Core_Team, 2012).

### Specificity of supragranular versus diffuseness of infragranular layers

To analyze supra- and infragranular projection fields with respect to double injections in V1 or V4, the surface area of labeling was reconstructed from serial sections through the projection zones. Plotting means versus standard deviation (SD) of these surface areas indicated that SD increased monotonically with the mean. A Box-Cox analysis (Box and Cox, 1964; Venables and Ripley, 2002) was performed to assess whether a power transformation of the form:

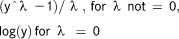
would render the variance more homoscedastic: the results suggested that a log transformation would be adequate. Finally, for the measures of projection field area, a linear mixed-effects model (Pinheiro and Bates, 2000) was used to estimate whether the differences in log area between the supra- and infragranular projection zones were significant.

### Estimation of hierarchical levels

In order to model hierarchy with SLN, we start with the counts of the number of neurons in the supra- and infragranular layers, (S, I)*_ij_*, projecting from a source area, i, to a target area, j. While the SLN is defined on the interval (0, 1) as the proportion of supragranular labeled neurons, SLN = S / (S + I), the vector representation as a pair of counts retains information on the strength of the projection. SLN is assumed to be a measure related to the hierarchical distance between two areas and the objective is to estimate a set of hierarchical levels that best predict the SLN values. A general model can be represented as a set of equations of the form:

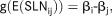
1where *β_i_* is the hierarchical value of area i (similarly for j) and g is a function linking the expected SLN value to the hierarchical distance between areas i and j. Considering a dataset of n projections involving a total of p source and target areas, a model for all of the data can be specified in matrix form as

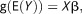
2where Y is a vector of length n containing the SLN values for each projection in the dataset (including possibly replicates of projections to the same area), β is a vector of length p containing the hierarchical levels to be estimated, and X is the n × p incidence matrix of the cortical graph revealed by the injections. An incidence matrix of a graph has one row for each edge and one column for each node of the graph. For the present dataset, it is constructed so that each row corresponds to a projection between two areas (e.g., V2 → V1, V4 → V1, etc.) and each column to an area (V1, V2, V3, etc.). All of the elements of a row are zero except in the two columns corresponding to the areas participating in the projection for that row, taking the values of −1 and +1 for source and target, respectively.

Assuming a probability model for SLN distribution and a link function, g, the hierarchical levels can be estimated via a maximum likelihood regression model, as shown below. The matrix is singular (each row adds to zero); in order to make the model identifiable, one column was therefore deleted, fixing the hierarchical level for the corresponding area at 0. The fitted hierarchy was arbitrarily normalized with respect to V1 in this fashion. The estimated hierarchy is only determined up to a linear transformation, however (adding a constant to each hierarchical value or multiplying by a constant will leave the predictions unchanged); therefore, estimated levels were transformed to a hierarchical scale varying from 1 to 10 for a more direct comparison of the present results with the FVE hierarchy.

The model as specified above is quite general. For example, if the link function, g, is taken to be the identity function, then Eq. 2 can be solved by least squares. This is equivalent to the approach of Barone et al. (2000), in which SLN differences were treated directly as hierarchical distances. Setting g to be a sigmoidal function of the SLN, such as log(S/I) (logit) or an inverse cumulative Gaussian (probit), would correspond to fitting a Generalized Linear Model (GLM) with a binomial family (McCullagh and Nelder, 1989). The laminar distribution of neurons would then be treated as a binomial variable taking on the value “success” for each supragranular neuron and “failure” for each infragranular neuron. Extending this idea, if the response, Y, is constrained to be a simple binary variable (e.g., Y = 0 if SLN <0.5 and otherwise 1), then this would correspond roughly to the approach taken by Felleman and Van Essen (1991) in their original article, in which they attempted to order the areas hierarchically to minimize the number of violations with respect to the binary ordering according to the laminar origin and termination of interareal projections.

We initially considered a binomial GLM to model the SLN values, but ultimately rejected it owing to the overdispersion of neural count data (Scannell et al., 2000; Markov et al., 2011, 2012). Instead, we used a beta-binomial model, which includes a dispersion parameter. The beta-binomial model, as its name suggests, is obtained as a mixture of beta and binomial distributions. The binomial parameter, p, corresponding to the proportion of successes, is considered to be a random variable following a beta distribution. The beta-binomial distribution can be formalized to depend on two parameters, μ and *φ,* that characterize its mean (corresponding to the probability of success or, here, SLN) and dispersion, respectively. In this parameterization, its likelihood is written as:

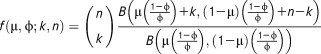
3where in the current context k is taken to be the number of supragranular counts for a projection, n the total number of counts, and B the beta function defined as:

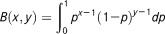
4with x, y > 0. The variance of the estimated proportion is (1/*n*)μ(1 − μ)(1 + (*n* − 1)*φ*). In fitting the model, we set μ = Φ(Xβ), where Φ is a cumulative Gaussian, since it maps the real line onto the interval (0, 1), and Φ^−1^ = g from Eq. 2 is a probit link function. The log likelihood of the model over the dataset is defined as:

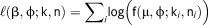
5Then, the hierarchical levels in the vector β and the dispersion *φ* can be estimated using an optimization routine that minimizes the negative of ℓ. Standard errors for each parameter are obtained from inverting the Hessian matrix, the second derivative of ℓ with respect to all pairs of the estimated parameters, evaluated at the maximum likelihood solution, and taking the square roots of its diagonal elements.

In practice, we fitted the beta-binomial model to our data using the function *betabin* from a version of the aod package (Lesnoff and Lancelot, 2012) in the OpenSource software R (R_Core_Team, 2012) that we had modified so that it could use a probit link for the function g. The function fits a beta-binomial model to a dataset by maximum likelihood. It uses an optimization routine based on the Nelder-Mead algorithm (Nelder and Mead, 1965) that, while not particularly fast, is nevertheless robust. The default value, logit, gives very similar results to the probit link. We chose the probit link so that the results would be comparable to other modeling that we had performed.

### Anatomical distances

Distances used in the present study (Table 2) were measured through the white matter in a 3D reconstruction of the M132 brain atlas and were measured between geometric centers of cortical areas (Markov et al., 2013).

### Photomicrographs

The initial color image in Figure 2 has been rendered as a grayscale. In Figures 2 and 1 contrast and brightness were adjusted in Adobe Photoshop CS5 (San Jose, CA). The file was subsequently exported to Adobe Illustrator CS5 so that laminar limits could be indicated.

## RESULTS

The present study defines pathways as FF or FB according to their SLN value: pathways that predominantly have their parent neurons in the supragranular layers are termed FF, and those in the infragranular layers are termed FB. The FVE model defines a category of “lateral” connections (Felleman and Van Essen, 1991), postulated to exist between areas on the same hierarchical level, displaying approximately equal numbers of parent neurons in the supra- and infragranular layers and terminating in all layers of the target area. The present study does not involve a category of lateral connections.

### SLN constrains the hierarchical organization of early visual areas

Cortical target areas were found to receive projections from between 34 to 87 source areas (Markov et al., 2012). For each injection, the fraction of labeled neurons in a given area with respect to the total number of labeled neurons in the cortical hemisphere identifies the FLN, which serves as a weight index (Markov et al., 2011). The laminar distribution of retrogradely labeled neurons in source areas defines the SLN (see Fig. 1 for more details on estimation of SLN and FLN). SLN was proposed to indicate the hierarchical distance of a source area. FLN could, in addition, enable the SLN values to be weighted. Here we explore mathematically the meaning of SLN as an indicator of hierarchical distance and use this index to constrain the cortical hierarchies.

**Figure 3:**
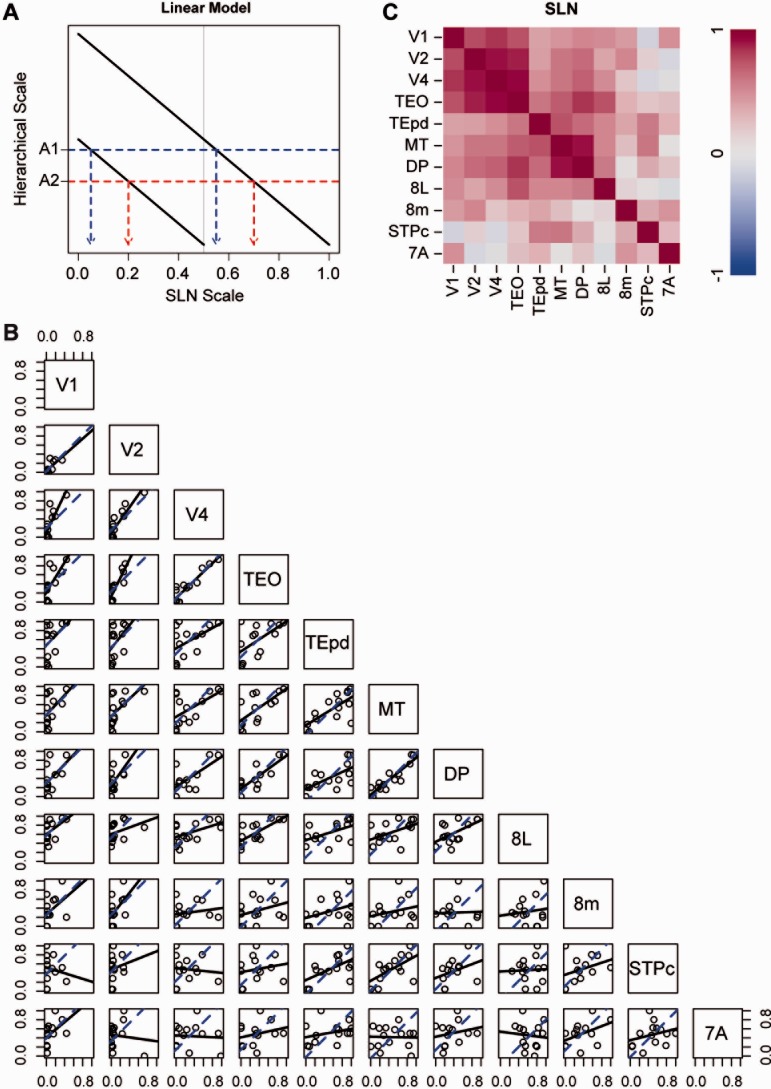
A: Schematic illustration of linear model of relation of SLN to hierarchical level. Relative hierarchical scale values depend directly on the difference of SLN values. For a given injection in two hypothetical areas, hierarchical distance is a linear function of SLN. The difference in hierarchical level maps onto a fixed SLN difference in each injection, indicated by the difference between each red dashed and blue dotted line as projected on the SLN scale axis for the hypothetical areas A1 and A2. B: Pairs plots: a set of scatterplots showing the correlation between SLN values obtained in common source areas labeled from specific pairs of injected areas (as indicated along the diagonal). Each point represents the average pair of SLN values obtained in a single source area. The blue dashed lines are the “best fit” lines of unit slope. The solid lines are the “best fit” lines that dually minimize distance from the points in both axes. C: Correlation matrices from the pairs plots of raw SLN values.

The working hypothesis was that SLN provides a measure of hierarchical distance between areas in the cortex (Barone et al., 2000). The strong version of this hypothesis would state that the difference between the SLN values obtained between two given areas will be identical, independently of injection site. Under this hypothesis, SLN is considered as a rigid ruler that can be shifted to have a reference point at the injection site (Fig. 3A). Wherever it is shifted, the difference between SLN values remains unchanged. The prediction of this hypothesis is that when SLN values from common source areas to different injection sites are plotted against each other, they will fall along a line of unit slope. Figure 3B shows a set of pairs plots for the SLN values from 11 injection sites (indicated along the diagonal), based on average data when multiple injections were available. Averages were obtained by adding supra- and infragranular counts across injections and computing SLN values on the totals. This procedure weights the contribution of each projection by its size. Points can only be plotted when a pair of areas are both targets of a common source area. While some of the plots are quite noisy, the ventral stream areas, and also MT and DP, seem to show a general correlation in the scatterplots. The strong version of the SLN hypothesis corresponds to the blue dashed lines, providing the best fit to unit slope. The degree to which the hypothesis is supported can be gauged by comparing these lines with the solid black lines that are best linear fits with no constraint on the slopes. The correspondences between the two lines are generally poor for injections in the higher-order areas, 8L, 8m, STPc, and 7A.

The solid lines in Figure 3B actually correspond to a slightly weaker hypothesis, that SLN differences measure the hierarchical distance from each injection site, but that the ruler might be stretched or contracted with respect to different injection sites. In the schema of Figure 3A, the lines indicating the relation between SLN and hierarchical level would not all be of the same slope. This hypothesis predicts that the relation between SLN values obtained from different injection sites simply follows a straight line with an unspecified slope. The degree to which this weaker hypothesis holds can be evaluated by examining the correlation of SLN values for each pair of areas, shown in Table 3, and the map of correlations in Figure 3C. As shown in Figure 3B, areas that exhibit a strong agreement between the two lines also tend to display high correlations. The range of correlations in Table 3 is (−0.15, 0.92) with mean of 0.43 (median = 0.47, interquartile range = (0.25, 0.61)). If we restrict the range of areas considered to the five ventral stream areas, MT and DP, the mean increases to 0.67 (median = 0.68, interquartile range = (0.58, 0.82)).

**Table 3:** Correlation Between SLN Values

	V1	V2	V4	TEO	TEpd	MT	DP	8L	8m	STPc	7A
V1	1.00	0.76	0.82	0.72	0.39	0.45	0.51	0.50	0.42	−0.15	0.47
V2	0.76	1.00	0.90	0.86	0.40	0.59	0.63	0.37	0.51	0.15	−0.10
V4	0.82	0.90	1.00	0.92	0.48	0.59	0.68	0.50	0.19	−0.10	−0.06
TEO	0.72	0.86	0.92	1.00	0.58	0.69	0.82	0.73	0.31	0.19	0.25
TEpd	0.39	0.40	0.48	0.58	1.00	0.72	0.64	0.51	0.36	0.57	0.30
MT	0.45	0.59	0.59	0.69	0.72	1.00	0.90	0.51	0.25	0.58	−0.03
DP	0.51	0.63	0.68	0.82	0.64	0.90	1.00	0.57	0.04	0.33	0.21
8L	0.50	0.37	0.50	0.73	0.51	0.51	0.57	1.00	0.12	0.04	−0.09
8m	0.42	0.51	0.19	0.31	0.36	0.25	0.04	0.12	1.00	0.34	0.45
STPc	−0.15	0.15	−0.10	0.19	0.57	0.58	0.33	0.04	0.34	1.00	0.28
7A	0.47	−0.10	−0.06	0.25	0.30	−0.03	0.21	−0.09	0.45	0.28	1.00

**Figure 4:**
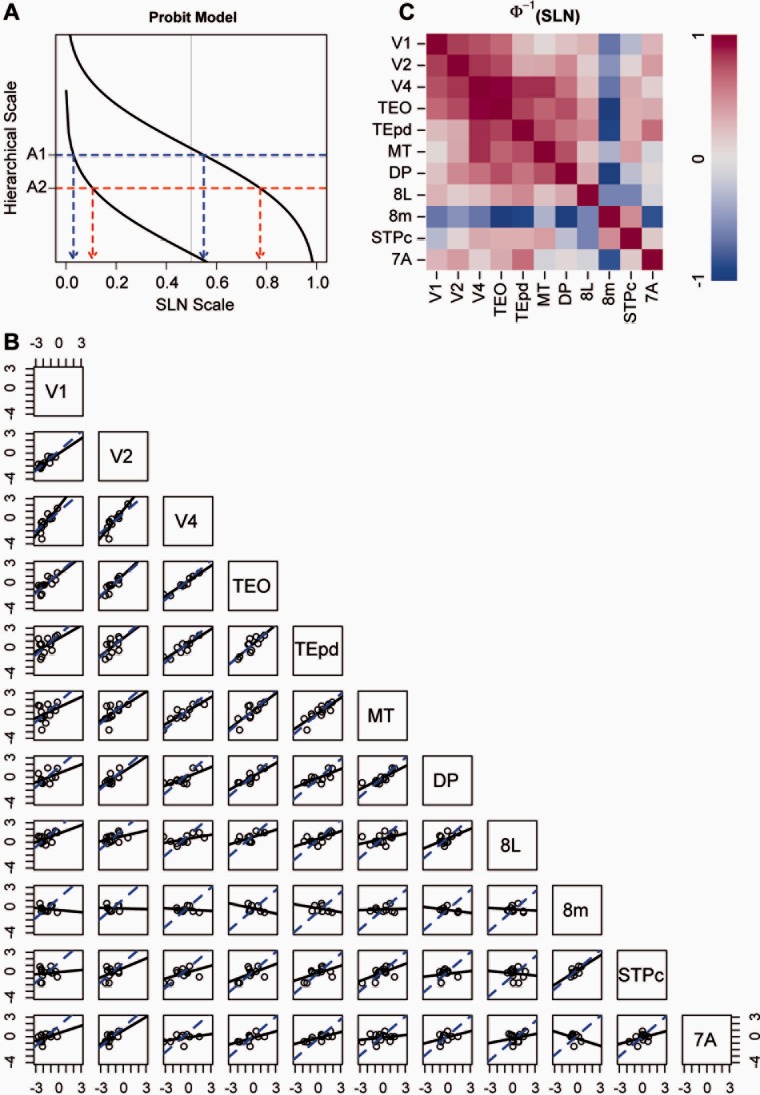
A: Schematic illustration of probit model of the relation of SLN to hierarchical level. Hierarchical scale values depend on SLN values through a sigmoidal transformation, here given by a probit (inverse cumulative Gaussian) transformation. A fixed hierarchical distance between hypothetical areas A1 and A2 corresponds to different SLN differences, depending on the injection. Conversely, small differences near extreme FB or FF values (0 and 1, respectively) can translate into the same hierarchical distances as larger SLN differences for more lateral connections. B: Pairs plots between probit-transformed SLN values of common areas from different injections. Conventions are otherwise the same as for the pairs plots in Figure 3B. C: Correlation matrices from the pairs plots of probit transformed SLN values. Ventral stream areas display high positive correlations, which seem to be accentuated by the probit transform.

SLN has some disadvantages as a measure of distance, in that it is restricted to the interval (0, 1). For example, the variance for variables in a fixed range is not usually distributed uniformly. This results in points near the ends of the interval contributing to the estimated hierarchical relation differently than those in the middle. In addition, note that, in Figure 3A, mapping from SLN to hierarchical level is truncated for one of the injections by the limits of the SLN range (0, 1). To deal with this, variables restricted to a unit interval are often transformed to the real line by a sigmoidal function such as the logit or the probit. A schematic demonstration of probit mapping from SLN to hierarchical level is shown in Figure 4A. The function relating SLN to hierarchical level approaches the limits asymptotically, so long distances are foreshortened but the SLN differences for equal hierarchical distance between a pair of areas depend on the distance they are from the injection site. Equal hierarchical distances do not necessarily map into equal SLN differences. This type of transformation has the advantage that it often renders the variance more homogeneous. For example, Fig. 4B shows the pairs plots for SLN on a probit scale. The sets of areas for which the two lines of best fit in each panel are nearly coincident has not changed, but the scatter in the data around the linear trends has been visibly reduced and some of the differences between the two fits have been lessened (e.g., for the two plots on the row with V4 and the three with TEO). The higher-order areas, however, show little evidence of a hierarchical relation, with the exception of the plot for the pair 8m and STPc. Interestingly, neighboring areas 8L and 8m show no evidence of correlation with respect to their inputs. And, while not all correlations have increased in the correlation matrix for the transformed SLN values in Table 4, for the seven areas that were identified above as showing the strongest agreement with the prediction of hierarchy, the tendencies of the correlation increased (mean = 0.73, median = 0.81, interquartile range = (0.65, 0.84)). This is also visible in the correlation map obtained from these data (Fig. 4C).

**Table 4:** Correlation Between Probit-Transformed SLN Values

	V1	V2	V4	TEO	TEpd	MT	DP	8L	8m	STPc	7A
V1	1.00	0.85	0.81	0.75	0.47	0.35	0.44	0.50	−0.17	0.11	0.49
V2	0.85	1.00	0.87	0.83	0.52	0.55	0.65	0.39	−0.05	0.36	0.58
V4	0.81	0.87	1.00	0.96	0.89	0.88	0.72	0.46	−0.17	0.52	0.37
TEO	0.75	0.83	0.96	1.00	0.84	0.77	0.82	0.58	−0.40	0.51	0.52
TEpd	0.47	0.52	0.89	0.84	1.00	0.82	0.71	0.65	−0.34	0.51	0.73
MT	0.35	0.55	0.88	0.77	0.82	1.00	0.81	0.44	0.08	0.57	0.23
DP	0.44	0.65	0.72	0.82	0.71	0.81	1.00	0.54	−0.38	0.13	0.35
8L	0.50	0.39	0.46	0.58	0.65	0.44	0.54	1.00	−0.11	−0.11	0.23
8m	−0.17	−0.05	−0.17	−0.40	−0.34	0.08	−0.38	−0.11	1.00	0.63	−0.28
STPc	0.11	0.36	0.52	0.51	0.51	0.57	0.13	−0.11	0.63	1.00	0.42
7A	0.49	0.58	0.37	0.52	0.73	0.23	0.35	0.23	−0.28	0.42	1.00

**Figure 5:**
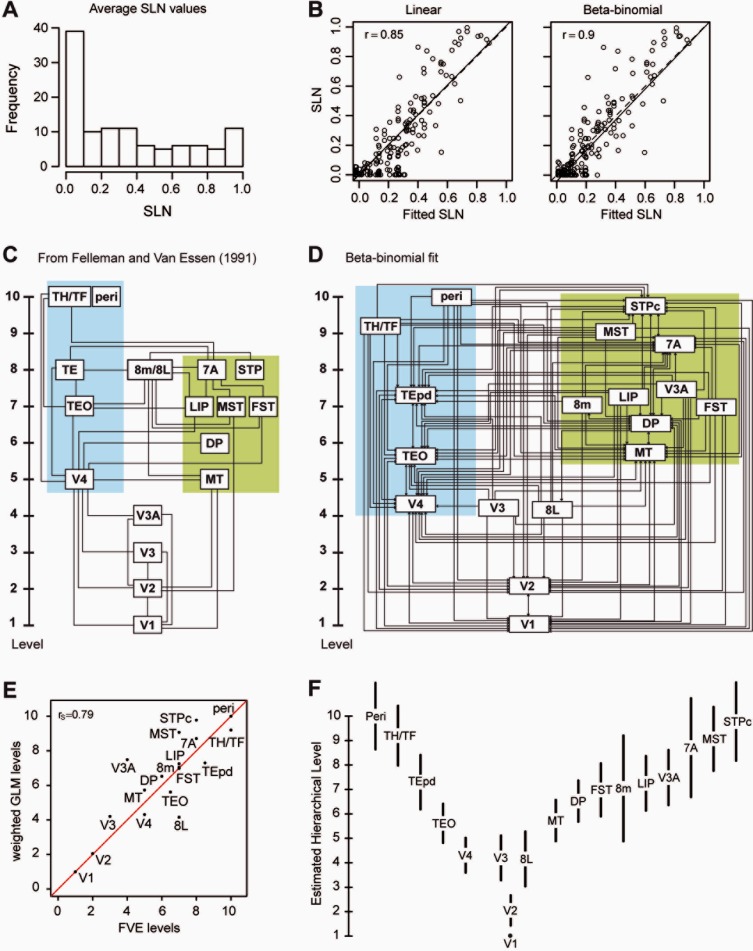
A: Frequency distribution of SLN values. B: Relation between the observed and predicted SLN from the linear and beta-binomial models C: Model of hierarchy of visual areas derived from Felleman and Van Essen (1991). D: Model of hierarchy of visual areas built form SLN and FLN values shown in Table 2. Blue background ventral stream areas; green background dorsal stream areas. E: Correlation of the hierarchy shown in (C,D). F: Estimated hierarchical levels from the beta-binomial model with 95% confidence intervals for the estimated values.

Figure 5A shows the histograms for the distribution of averaged SLN values for the seven target areas with the strongest evidence of a consistent hierarchical relation according to the pairs plots. Aside from the large contingent of FB connections at SLN values below 0.1, the distribution is approximately uniform, with no obvious evidence of clustering. This is consistent with the presence of a continuous spectrum of SLN values in the projections among the areas.

Using the raw SLN to estimate hierarchy is equivalent to fitting a linear model to the differential SLN values. This was compared with a model in which the hierarchical values were related to SLN through a probit transformation. A beta-binomial model was used in this latter case because it allowed an estimation of overdispersion in the data to be included. We also tried to fit the data with a binomial GLM for both SLN as a binomial count and for a binary variable indicating whether the SLN value was of an FB or FF type (not shown). The fixed dispersion of the binomial GLM and the large number of counts for many of the projections led to extremely small and probably unrealistic standard errors being estimated. On the other hand, the use of a purely binary response variable for SLN produced huge standard errors, indicating great indeterminacy in the hierarchy estimated in this fashion.

Each of the models estimates the hierarchical values that best predict the SLN values according to a particular criterion. Goodness of fit was assessed in terms of how well the fitted SLN values predicted the experimental values, shown in the scatterplots of Figure 5B. Both models displayed a strong positive correlation between the fitted and experimental SLN values. There appeared to be a tendency in the linear model for the fitted FF values to underestimate the true SLN values (SLN > 0.5) and to overestimate FB values (SLN < 0.5), which was less marked using the beta-binomial model. The solid line gave the best fit, while the dashed line is the line of unit slope through the origin. The difference between these two lines was smaller for the linear model. Nevertheless, the correlations (shown for both graphs in the upper left corner) indicated that the beta-binomial model predicted SLN values significantly better than did the linear model (*z* = 2.15, *P* = 0.03).

Figure 5C and D compare the FVE qualitative hierarchy and that based on the beta-binomial SLN. Figure 5E shows that there is a good correlation between the two hierarchies. An important difference between the FVE hierarchy and the model based on quantitative measurements concerns the position of the 8L component of the frontal eye field. In the FVE model, the frontal eye field is located on level 8, at the same level as TE, 7A, and STP. In the quantitative hierarchy, the frontal eye field is separated into two components, 8L and 8m. Whereas 8m remains at the same hierarchical level as 8m in the FVE model, 8L is at the same level as V4 in the present model. Cluster analysis (not shown) suggested that 8m is more integrated into the dorsal than the ventral stream.

Despite the high correlation between the fitted and observed SLN values, one may wonder about the variability of the estimated hierarchical positions. Figure 5F shows the hierarchical estimates with 95% confidence intervals obtained from the variance-covariance matrix of the fit; these vary between ±1 and ±2 hierarchical levels. By design, area V1 was fixed at level 1 and therefore shows no variability.

### Weight-distance relations and hierarchy in the visual cortex

**Figure 6:**
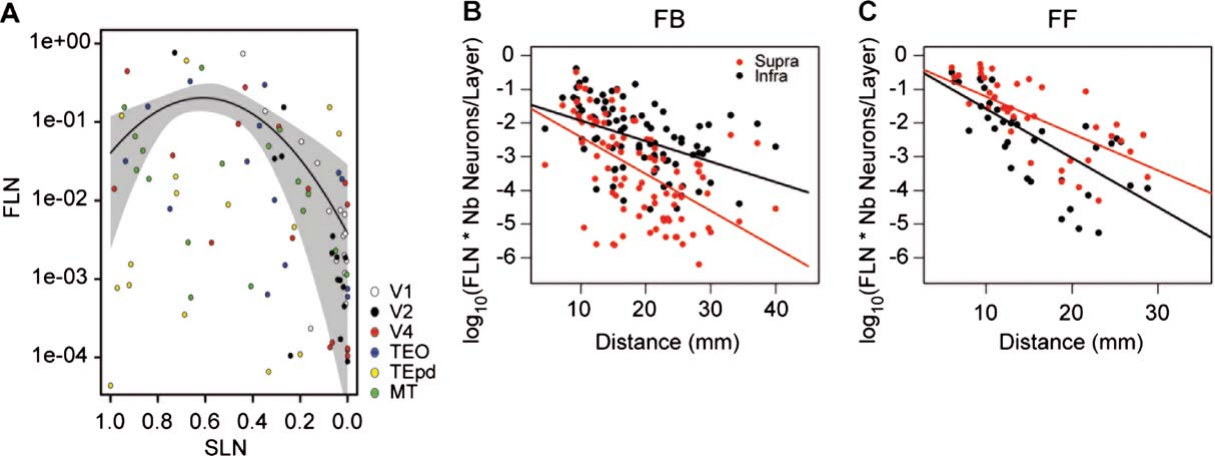
Combinatorial distance rule determines the SLN of FF and FB projections. A: Relationship of FLN to SLN. The curve is the best fitting parabola and the gray envelope indicates the standard errors of the fit. B: FB projections, fit with a linear model to the supra- and infragranular layer fractions of the FLN. This figure is generated based on injections in nine areas (V1, V2, V4, DP, TEO, TEpd, STPc MT, and 7A).The prefrontal areas were excluded from the source and target list due to their tendency to overrun the distance and hierarchy rules. The lines are constrained to have a common intercept at the origin. The constrained fit did not differ significantly from an unconstrained fit in which independent intercepts were permitted (F(1, 172); 0.40; *P* = 0.53). C: Same analysis as in (B) for the FF projections. Again, constrained fit did not differ significantly from unconstrained fit (F(1, 74) = 0.86, *P* = 0,31).

Elsewhere we have shown that the properties of the cortical network are shaped by the decline in connection weight over distance (Ercsey-Ravasz et al., 2013; Markov et al., 2013). In the FVE model, cortical hierarchy is found to match the physical layout of the cortex relatively well, with rostral directed connections going up the hierarchy and caudal directed connections going down. In the SLN model, the relation between hierarchical distance and physical distance generates the hierarchy shown in Figure 5D. The exponential decline of connection weight with distance (Ercsey-Ravasz et al., 2013) leads to prediction of a relationship between SLN and FLN, which was indeed the case, as shown in Figure 6A. This figure shows that projections with SLN around 0.5 exhibited the highest FLN values, which declined toward SLN values of 1.0 and 0.0. Hence, the SLN values nearing 50%, as in areas V2 to V1, correspond to short distances and high values, as in V4 to V1, to long distances. Lateral connections and connections between adjacent levels tend to cover short distances across the cortex and have high FLN values, whereas connections that cross multiple levels correspond to longer physical distances and low FLN values (Fig. 6A).

The fact that SLN indicates hierarchical distance means that for FF pathways there is a steady decline with distance in the proportion of infragranular projection neurons in the FF direction. Conversely, for FB pathways there is a similar decline in the proportion of supragranular neurons in the FB direction. Here we address the question as to whether these decreases reflect rules governing the projection distances of these two sets of neurons in FF and FB pathways.

The above considerations suggest that the projection distances of neurons in individual layers could depend on whether they are projecting up or down the cortical hierarchy. For instance, it might be predicted that supragranular layers project for long distances in rostral directions and short distances in caudal directions. To explore this possibility, we analyzed the SLN fractionated FLN (SLN * FLN and (1 − SLN) * FLN). That is, the fraction of FLN attributed to supra- and infragranular layer neurons as a function of the physical distance traversed by the projection (for distance measures, see Materials and Methods). This analysis was carried out separately for the FF (*n* = 39) and FB (*n* = 88) pathways of areas V1, V2, V4, TEO, TEpd, MT, DP, STPc, and 7a. This measure, rather than the raw numbers of neurons, adjusts each injection for the total number of neurons counted. In all cases, there was a decrease in the fraction of labeled neurons with increasing distance. For FB projections (Fig. 6B), the slope for the supragranular layers was significantly steeper than for the infragranular layers (F(1, 173) = 37.4, *P* < 0.001). In the case of the FF projections (Fig. 6C) the situation was reversed, with the infragranular layer slope being significantly steeper (F(1, 75) = 8.92, *P* < 0.01).

These results show that, in extrastriate cortex, infragranular layer FB neurons project significantly further than do the supragranular layers and these two sets of space constants are directly responsible for the SLN values of FB pathways. In contrast, the supragranular FF neurons project for significantly longer distances than the infragranular layers, giving rise to the FF SLN signature.

### Incidence and magnitude of FF and FB pathways

**Figure 7:**
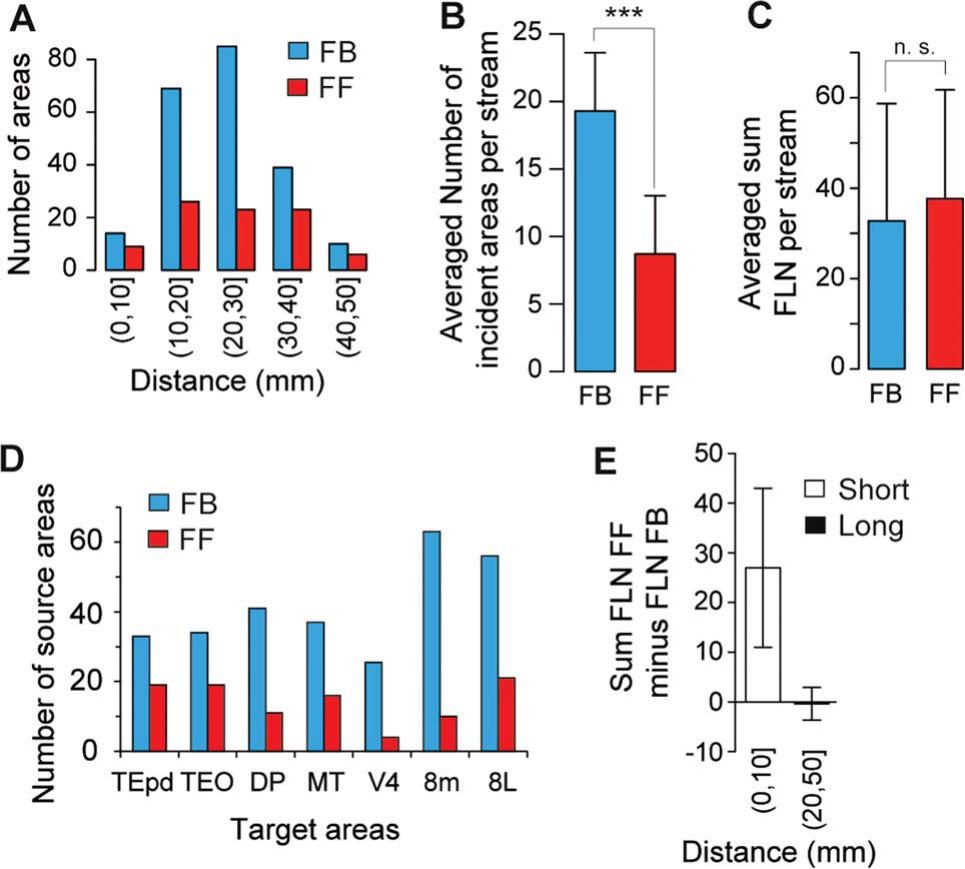
Influence of distance from target area on FF and FB pathways (target areas V1, V2, V4, DP, MT, TEpd, TEO, STPc, 7A, 8L, 8m). A: Incidence of FF (100% ≥ SLN% ≥ 55%) and FB (0% ≤ SLN% ≤ 45%) at different distance intervals. B: Comparison of the average numbers of FF and FB pathways for each target area. C: Average across injections of the sum of FLN in FF and FB pathways. D: Incidence of FF and FB in middle hierarchy areas. Conventions as in (A). E: Influence of distance on FLN magnitude. For each target area we subtracted the sum total FLN% of FB projections from the sum total FLN% of FF projections. The histogram represents the median of the result. Error bars: median absolute deviation, short distance 0–10 mm, long 20–50 mm.

Several anatomical studies suggested that FB pathways are more frequent than FF pathways (Perkel et al., 1986; Salin and Bullier, 1995). The present study quantified the frequency and magnitude of FF and FB projections in the 339 pathways of the visual cortex projecting to areas V1, V2, V4, DP, MT, TEpd, TEO, STPc, 8L, 8m, and 7A. This revealed that there were twice as many FB pathways as FF pathways (Fig. 7A,B). However, this predominance of FB pathways is tempered when the relative weights of the pathways are taken into account. On average, the cumulative FLN of projections involved in the FF pathways to a given area is not significantly different than the cumulative number of neurons involved in the FB pathways (Fig. 7C). This observation suggests that the average weight of FF exceeds that of FB, given that FB pathways are more numerous than FF pathways (Fig. 7A,B,D). Figure 7E compares summed FLN values at short (0–10 mm) versus long (20–50 mm) distances: distance has an important influence on the cumulative strength of these pathways. Normalizing the numbers of neurons labeled after each injection allows correction for injection size differences and comparison between the relative investment of neural resources in each pathway. FF projecting neurons dominate over short distances, and FF and FB are about equal over long distances; hence, on average nearly 80% of neurons projecting less than 10 mm participate in an FF pathway, while on average 60% of neurons engaged in projections longer than 10 mm are in an FB pathway.

It could be objected that the observed preponderance of FB pathways in the visual system reflects a bias caused by inclusion of areas V1 and V2. This is not in fact the case: as shown by Figure 7D, the areas in the middle region of the hierarchy (TEpd, TEO, DP, MT, V4, 8m, and 8L) were also dominated by high numbers of FB pathways.

### Topography of FF and FB projections

Here we investigated whether topological precision is a characteristic of the layer or the pathway. There have been numerous claims that FB projections exhibit higher bifurcation frequencies and are more diffuse than FF projections (Salin and Bullier, 1995). If supra- and infragranular layer neurons show marked differences in topographical precision, then the reported differences in FF and FB pathways could stem from differences in the relative contributions of the upper and lower layers (i.e., supragranular being preponderant in FF and infragranular in FB projections). To examine if this was the case, we measured the respective topographical precision of both sets of layers in FF and FB pathways.

Previous studies showed that topographical precision can be investigated by making side-by-side injections with tracers such as those used in the present study (Fast blue and Diamidino yellow), which are readily distinguishable in retrogradely labeled neurons and have been shown to have restricted and clearly defined uptake zones (Perkel et al., 1986; Salin et al., 1989, 1992; Kennedy et al., 1994). Dual injection in the target area produced two populations of retrogradely labeled neurons in the source areas, where the degree of overlap of the two populations reflects the interinjection separation as well as the topographical precision of the connections between the source and target areas. Within the region of overlap there was a small population of double-labeled neurons: neurons with collaterals targeting both injection sites (Kennedy and Bullier, 1985). The dimensions of the overlap zone and the number of double-labeled neurons were used to gauge the topographical precision of the projection neurons in the source area.

Side-by-side injections of Fast blue and Diamidino yellow were made in areas V1 and V4. We measured 1) the spatial extent of the projection zones to each injection, 2) the spatial extent of the overlap of the projection zones, and 3) the percentage of double-labeled neurons in the overlap zones, thereby making it possible to compare the topographical precision of projection neurons in both sets of pathways (Perkel et al., 1986; Barone et al., 2000).

**Figure 8:**
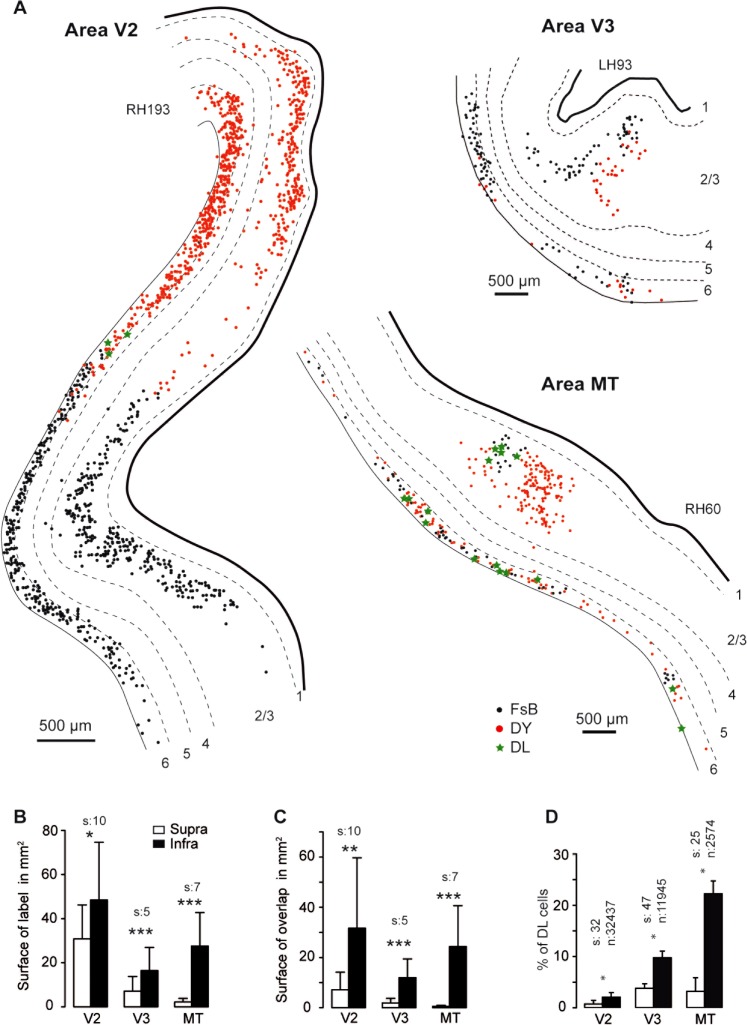
Topography of projections to area V1. A: Spatial layout of FB neurons in supra- and infragrauluar layers of extrastriate areas following dual injections in area V1. B: Histograms showing surface area of projection zones. C: Overlap surface of the projection zones of both dyes. D: Percentage of double-labeled neurons in overlap zone in B. FsB, Fast blue; DY, Diamidino yellow; DL, double labeled; s = numbers of sections, *n* = number of neurons. ***P 0.001, **P 0.01, *P 0.05.

Area V1 injections were analyzed in three animals over three source areas (V2, V3, MT); there were 18 observations of the response variable, which was the difference between the logs of the reconstructed projection zone surfaces (supra vs. infra) (Fig. 8). Analysis showed the infragranular projection zones to be significantly larger (on average by a factor of 12) than the supragranular projection zones (F(3, 13) = 74.7, t(8) = 6.47, *P* < 0.0001).

**Figure 9:**
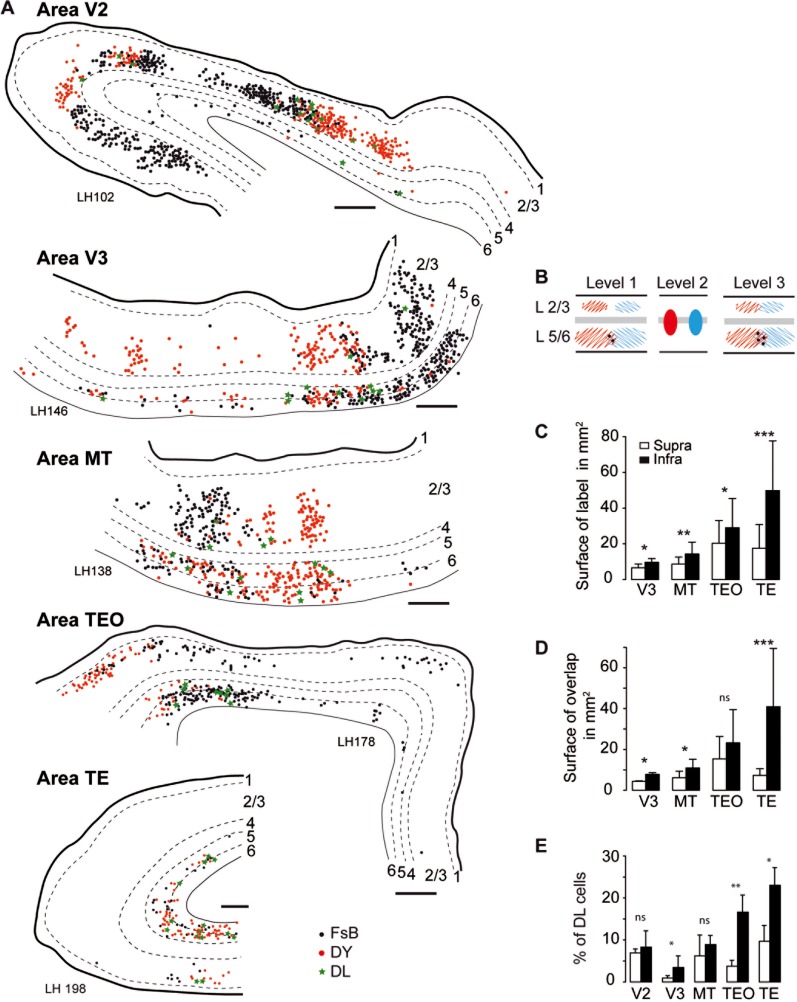
Spatial extent, overlap, and proportions of double-labeled neurons in extrastriate areas following dual injections of area V4. A: Charts of labeled neurons in extrastriate areas following dual injections in area V4. B: Schematic representation. C: Surface area in mm^2^ (number of sections for reconstructions: V3 = 6, MT = 9, TEO = 4, TE = 6). D: Surface area in mm^2^ of the overlap zone of FB and DY labeled neurons (number of sections as in C). E: Percentage of double-labeled neurons (V2 number of sections = 58, neurons = 13,231; V3 sections = 12, neurons = 6773; MT sections = 13, neurons = 3352; TEO sections = 3, neurons = 1971; TE sections = 5, neurons = 2291). Scale bar: 500 μm. FsB, Fast blue; DY, Diamidino yellow; DL, double labeled; empty bars supragranular layers, filled bars infragranular layers. ***P 0.001, **P 0.01, *P 0.05.

Figure 9 shows the analysis of the area V4 injections carried out in three animals over five source areas (V2, V3, MT, TE, TEO) and, except for area V2, analyzed in the same manner as for the area V1 injections in Figure 8. For projection from area V2 to area V4, the patchy distribution of labeled neurons (DeYoe and Van Essen, 1985) and the sparse labeling in infragranular layers in this long-distance FF pathway made surface measurement comparisons impractical. Nevertheless, as shown in Figure 9A, the V2 projection to V4 showed marked segregation of the two populations of labeled neurons, reflecting interdigitation of point-to-point connectivity. For the other four projections, the infragranular projection zones were significantly larger (on average by a factor of 2) than the supragranular projection zones (t(11) = 4.83, *P* < 0.0001).

This confirms earlier findings that FF supragranular layers exhibit point-to-point connectivity; quantitative comparison show that this property is very similar to the point-to-point connectivity observed in the supragranular layer of the six FB pathways. Hence, irrespective of whether a pathway was FB or FF, the spatial extent and degree of overlap of projection zones and the frequency of double-labeled neurons were significantly higher in infragranular than in supragranular layers (Figs. 9). Thus, the convergence and divergence and the rate of bifurcation of cortical projections from infragranular layers is higher than for supragranular layers, independently of whether a pathway is FF or FB. These results suggest that it is the differential contribution of these layers to the two sets of pathways that largely defines the topographical precision of the cortical streams.

### Segregation of FF and FB projecting neurons

**Figure 10:**
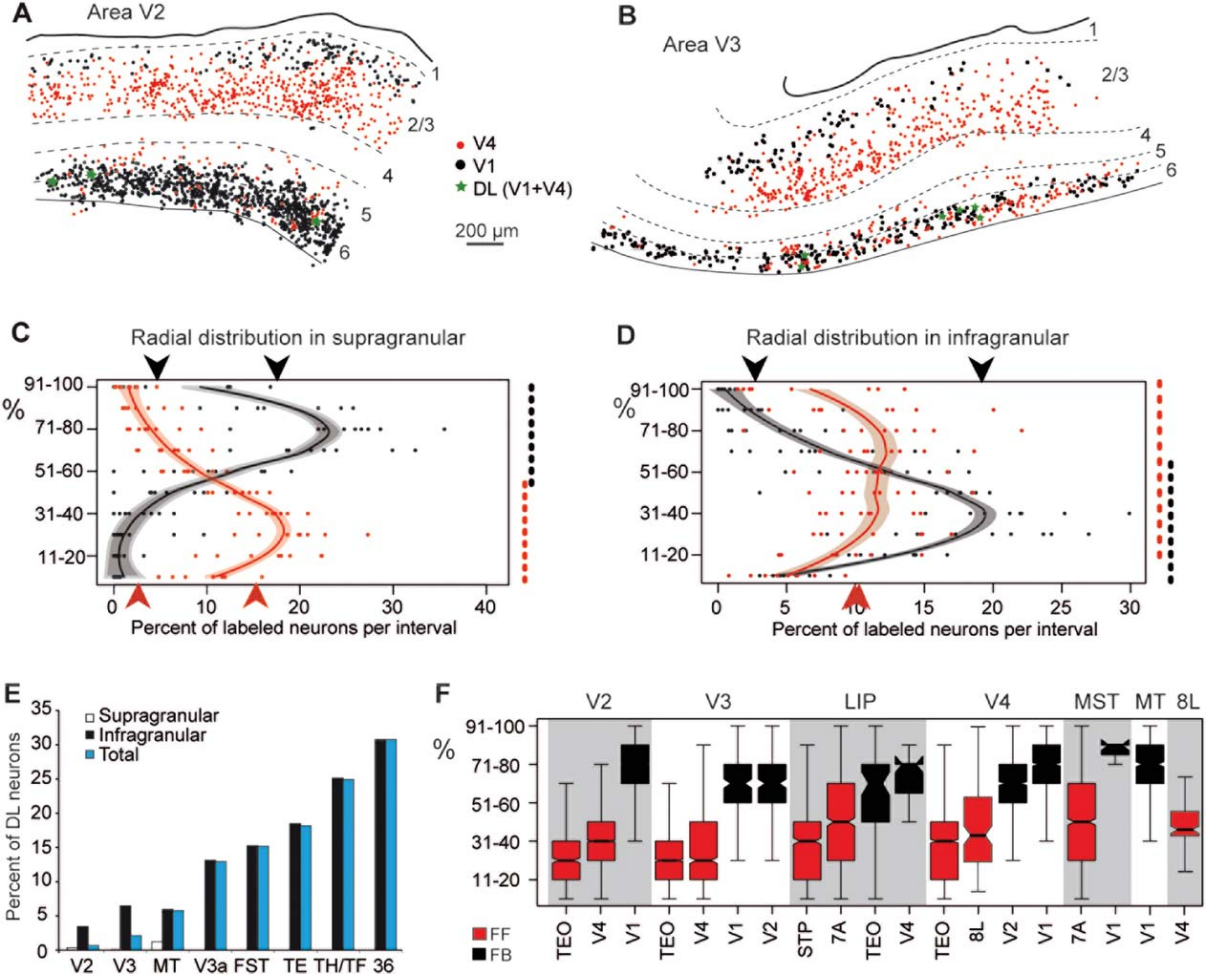
Segregation of FF and FB pathways. A,B: Charts of retrograde labeled neurons in a parasagittal section of area V2 (A) and area V3 (B) following injections of DY in area V1 and FsB in area V4. C: Percentage of labeled FF and FB neurons per depth bin in supragranular layers of extrastriate cortex (areas V2, V3, V4, LIP, MST, MT). Envelope corresponds to a loess predicted distribution. Black dashed line indicates the FB compartment identified by a tree model; the high and low mean for neuronal distribution within and outside the compartment are indicated by black arrowheads. Red dashed line identifies the FF compartment isolated by tree model, the red arrowheads indicate the high and low mean for neuronal distribution within and outside the compartment. D: The segregation of FF and FB neurons in the infragranular layers. Same conventions as in (C). E: Laminar distribution of double labeled neurons in visual areas V2, V3, V3a, MT, FST, TE, TH/TF, area 36. Proportions of double-labeled neurons expressed as percentages of the smallest population of single-labeled neurons. F: Boxplots of the distribution of neurons in supragranular layers for individual projection pathways. C,F: Ordinate scale goes from 0 top of layer 4, 100 bottom of layer 1. D: 0 bottom of layer 6, 100 top of layer 5. DL, double-labeled.

Early reports on the connectivity of macaque extrastriate cortex provided suggestive evidence of a radial separation of rostral and caudal directed projections emitted from the supragranular layers (Rockland and Pandya, 1979; Tigges et al., 1981; Rockland, 1997). We approached this quantitatively by simultaneous injection of the two distinguishable retrograde tracers, Diamidino yellow in area V1 and Fast blue in area V4. The distribution of labeled neurons in V2 and V3 (Fig. 10A,B) shows that there was clear segregation of the two populations of projection neurons. In the supragranular layers, the population of FB projecting neurons targeting V1 were concentrated in layer 2/3A and appeared largely separated from the population of FF projecting neurons targeting V4 and concentrated in layer 3B. In the infragranular layers, the two populations were largely intermingled: FB neurons targeting V1 were located in layer 6 and the bottom of layer 5, while the FF neurons targeting V4 were found throughout layers 5 and 6 (Fig. 10A). However, because of the heterogeneous laminar distribution of corticocortical neurons in the projection zone of a given pathway, demonstrating pathway segregation requires high-frequency sampling throughout the projection zone (Batardiere et al., 1998). In order to explore the patterns of cells of origins quantitatively, we analyzed 19 FF and FB pathways that targeted areas at different hierarchical levels (areas V1, V2, V4, STP, TEO, and 8L). The loess curves in Figure 10C,D suggest that the segregation of FF and FB projecting neurons is a consistent feature across the cortex. Clear segregation into an FF and an FB compartment was observed in the supragranular layers (Fig. 10C,E). A regression tree model was used to determine whether there were two separate compartments for each stream (Breiman et al., 1984; Ripley, 1996). By minimizing the residuals between the average and the counts, the regression tree model indicated that the supragranular layer is split into an upper FB and a lower FF compartment at mid-depth (red and black dotted line to the right of Fig. 10C). The infragranular layers had FF neurons throughout the width of the compartment, while the FB neurons were much denser in the lower two-thirds (red and black doted lines to the right of Fig. 10D).

Simultaneous injection of Fast blue and Diamidino yellow at retinotopically corresponding locations in V1 and V4 is a means of exploring the relative integration of FB and FF projecting neurons in the local circuits of their source areas. In the supragranular layers of area V1, 2.6% of the FF projecting neurons targeting area V4 were double-labeled, revealing a local intrinsic axon collateral. This contrasted with a much higher percentage in V4, where 13.6% of the supragranular FB neurons targeting V1 possessed locally projecting collaterals. These results did not reflect the presence or absence of a local collateral, but rather the relative integration of the projection neuron in the local circuitry, although greater integration may reflect the abundance and, more probably, the spatial extent of local collaterals.

Paired injection in areas V1 and V4 revealed the incidence of neurons projecting simultaneously to both targets, and which were therefore double-labeled, and also made it possible to examine whether FF neurons possessed axon collaterals projecting to FB targets and vice versa. Areas V2 and V3 have an FF neuron population projecting to area V4 and an FB population projecting to area V1: it was therefore possible to examine if single neurons possessed axon collaterals projecting to both targets (Fig. 10F). Double-labeled neurons were found to be very rare in both areas (less than 1% in V2, and 2.2% in V3) and were largely limited to the infragranular layers. Note that in areas higher than V4, the two populations of neurons projecting to both V1 and V4 are infragranular FB projecting neurons, and here double-labeling increased from 6% to 30% with increasing hierarchical distance, as observed previously (Kennedy and Bullier, 1985; Sincich and Horton, 2005). These findings show that neurons projecting to areas V1 and V4 are highly segregated in areas where they constitute respectively FB and FF connections, but not in areas where both sets of neurons constitute FB connections.

### Cell morphology experiments

Integration of the FF and FB connections into the laminar structure of the cortex depends not only on the laminar location of the parent pyramidal soma but also on its dendritic arborization. It is thought that, whereas corticocortical neurons in the supragranular layers have apical dendrites extending to and forming tufts in layer 1, infragranular corticocortical neurons have slender apical dendrites that do not reach layer 1 (Lund et al., 1981; Katz, 1987; Hubener et al., 1990). Because long-range cortical projections are formed by only a minute fraction of cortical neurons (Lee and Winer, 2008; Markov et al., 2011), what is known about the cell morphology of upper and lower layer neurons cannot be extrapolated to that of the parent neurons of interareal pathways that reside in these layers.

**Figure 11:**
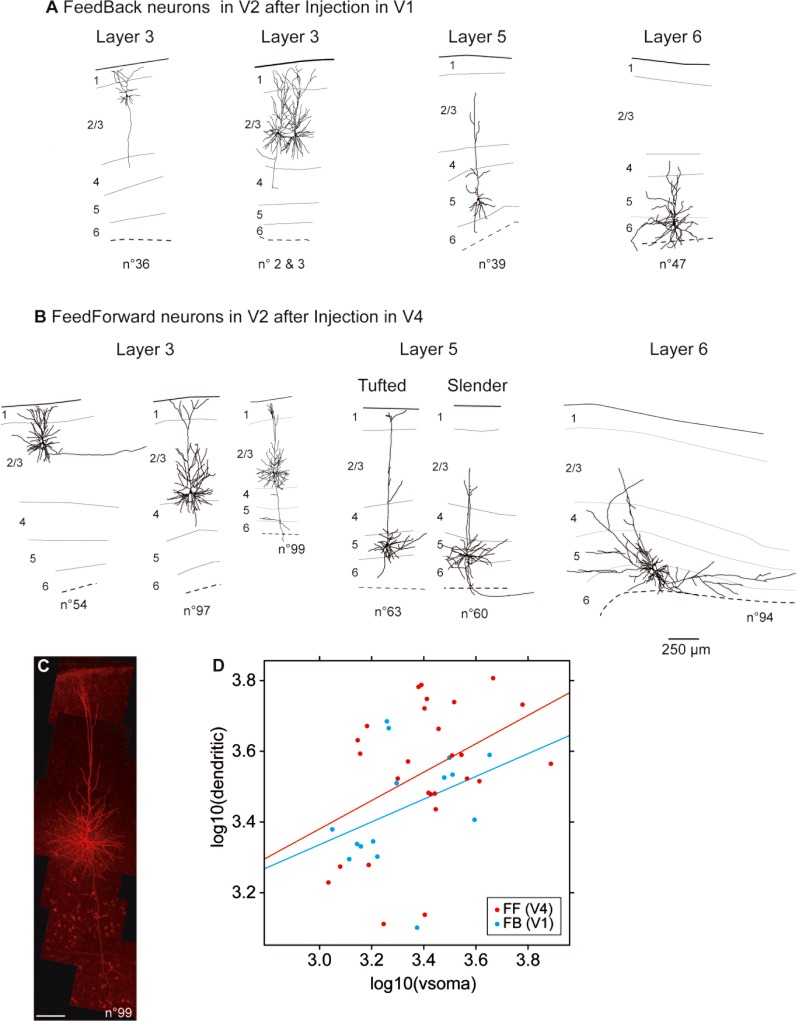
Morphology of projection neurons in V2. A,B: Area V2 cell morphology of parent neurons of interareal pathways. C: Photomontage reconstruction of an FF neuron in layer 3B shown in B. D: Scatterplot of cell soma size and dendritic arbors. Scale bars = 250 μm in A,B; 100 μm in C.

This issue was explored using retrograde tracers to identify both sets of neurons in area V2, and then performing ex vivo cell filling (see Materials and Methods). All of the 46 filled neurons that were recovered had clear pyramidal cell type morphologies with a well-defined apical dendrite (Fig. 1). The results showed that all supragranular layer neurons, whether FF or FB, possessed a tufted apical dendrite (first two panels Fig. 1A and first three of Fig. 1B). In the infragranular layers, FF and FB layer 6 pyramidal neurons (Fig. 1A, 4th panel; Fig. 1B, 6th panel) as well as FB layer 5 neurons (Fig. 1B, 5th panel) had slender apical dendrites, conforming to the morphology of corticocortical neurons previously described in these layers in rodents and cat (Klein et al., 1986; Katz, 1987; Hallman et al., 1988; Hubener et al., 1990; Kasper et al., 1994). Unexpectedly, four of the nine FF neurons in layer 5 had apical dendrites that reached layer 1, and three of these formed multiple branches in layer 1, conforming to the tall simple pyramidal neuron type found in contralateral corticocortical projections in mouse (Larsen et al., 2007) (fourth panel Fig. 1B). Dendritic filling was satisfactory and soma dimensions and extent of dendritic arbors were correlated; FF neurons were larger than FB neurons (Fig. 11D).

It is currently believed in carnivore and rodents that corticocortical layer 5 neurons are slender, while tufted layer 5 neurons target the superior colliculus and thalamus (Katz, 1987; Kasper et al., 1994; Tsiola et al., 2003; Larsen et al., 2007; Llano and Sherman, 2009). There is evidence in mouse that tall simple cells can form corticocortical connections (Larsen et al., 2007). The present results show monkey layer 5 neurons with apical dendrites that reach layer 1, corresponding to tall simple cells, which may be a characteristic feature of FF neurons in this layer. In the FB population, apical tufts were located only in the small contingent of FB neurons with short projections located in layer 2/3A, corroborated by previous studies showing an absence of apical tufts in the layer 6 FB neurons projecting to area V1 (Rockland and Virga, 1989; Rockland et al., 1994).

## DISCUSSION

### What are the FF and FB pathways?

Recent evidence suggests distinct neuronal dynamics in supra- and infragranular layers. Electrophysiological recordings show that supragranular neurons display predominantly gamma-band oscillation (Bollimunta et al., 2011; Buffalo et al., 2011; Xing et al., 2012), whereas infragranular layers display predominantly beta-band oscillation (Buffalo et al., 2011; Xing et al., 2012). This local synchronization can generate interareal synchronization (Buschman and Miller, 2007; Gregoriou et al., 2009; Salazar et al., 2012; Bosman et al., 2012), thought to be a mechanism of interareal interaction (Fries, 2005; Womelsdorf et al., 2007; Bosman et al., 2012). These observations suggest that the major stream in FF pathways promotes gamma synchronization, while the major stream in FB pathways promotes beta synchronization. This was tested by showing that a functional distance rule based on interareal Granger causal influences during attentional tasks correlated remarkably well with SLN (Vezoli et al., 2012).

The differences in the neuronal dynamics of supra- and infragranular layers provide additional evidence that these two compartments house pathways with distinct physiological roles. Conceivably, supragranular neurons in both FF and FB pathways have similar gamma-band oscillations, as well as anatomical properties (such as topography: see the present report, above). This contrasts with the infragranular layers, which show beta-band oscillation, again in both FF and FB. Accordingly, because of the change in SLN with distance, FB neurons projecting to adjacent and nearby areas have an important supragranular layer function, whereas FB projections to far distant targets have a small or nonexistent supragranular layer function. Similarly, FF projections with nearby targets have a relatively important infragranular layer function, which is considerably smaller or nonexistent for far distant projections.

### Cortical hierarchy

Globally, the ventral stream shows fewer differences from the FVE model than does the dorsal stream. There are a number of observations suggesting different hierarchical relations in ventral and dorsal streams (Schmolesky et al., 1998; Bullier, 2004; Chen et al., 2007).

Constructing a hierarchy in fact provides a global view of areal relations, which attempts to minimize distortions in the relative hierarchical positions of areas while maximally accommodating the SLN values of individual pathways. Figure 5D provides an overview of the interactions of the system, while the SLN values of a particular pathway may be more pertinent to understanding the interactions of the two interconnected areas concerned.

The FVE model used discreet levels in order to define their cortical hierarchy (Felleman and Van Essen, 1991). A number of studies have advocated using a continuous hierarchical scale (Kennedy and Bullier, 1985; Shipp et al., 1998; Barone et al., 2000; Batardiere et al., 2002; Vezoli et al., 2004; Grant and Hilgetag, 2005; Reid et al., 2009; Krumnack et al., 2010). Compared to models with discrete scales, continuous scales such as SLN may have the advantage of providing a more constrained set of hierarchical relations, thereby minimizing violations so as to select an optimized but not necessarily unique hierarchy (Krumnack et al., 2010).

Our earlier study found that the frontal eye field had a lateral and not an FB projection onto V4 (Barone et al., 2000), in line with other reports (Schall et al., 1995) and as subsequently confirmed (Ungerleider et al., 2008). The present study distinguished the 8L and 8m subdivisions of the frontal eye field, which correspond to the small and large saccade components (Schall et al., 1995; Gerbella et al., 2007; Markov et al., 2012). The present study shows that only area 8L is situated at the level of area V4, while 8m remains at the same level as in the FVE model. The present study goes considerably further than our earlier findings in a number of ways, placing 8L in a low position in the hierarchy. First, previously unknown projections of both V1 and V2 to area 8L have been demonstrated (Markov et al., 2012), thereby reinforcing the position of 8L at an early stage of the visual system. Second, the cumulative FLN of the connections of area 8L with ventral stream areas (data not shown), its connections with TH/TF and its central position in large-scale models of the cortex (Vezoli et al., 2004; Sporns et al., 2007) are anatomical arguments in favor of this prefrontal area occupying a central position in the ventral stream hierarchy (for an exploration of its possible central role in information processing, see below: “What are the processes supported by the FF and FB counterstreams?”). This relatively low location in the visual hierarchy is compatible with the possibility that area 8L drives attentional processes in area V4 (Anderson et al., 2011; Miller and Buschman, 2013). Overall, given the differences in connectivity and hierarchy, future studies will decide whether 8L and 8m are two subdivisions of a single area or constitute two distinct areas.

The present study examined the hierarchal organization of cortical areas purely in terms of the values of SLN. The laminar distribution of connections linking cortical areas is related to the architectonic differentiation of the source areas, in line with the fact that primary sensory areas have a well-defined layer 4 and can be expected to be a source of FF pathways. Hence, projections from eulaminate cortices with six well-defined layers show high proportions of parent neurons in supragranular layers, whereas projections from areas such as the limbic cortex, which show less well-defined lamination, tend to originate from infragranular layers (Barbas, 1986; Reser et al., 2013). Hence, the present findings suggest that changes in SLN may be related to the degree of differentiation of the interconnected areas as has been claimed in cat visual cortex (Hilgetag and Grant, 2010). It could be interesting to see how a combination of SLN and architectonic differentiation interacts to constrain cortical hierarchy in primates.

### Segregation of pathways

**Figure 12:**
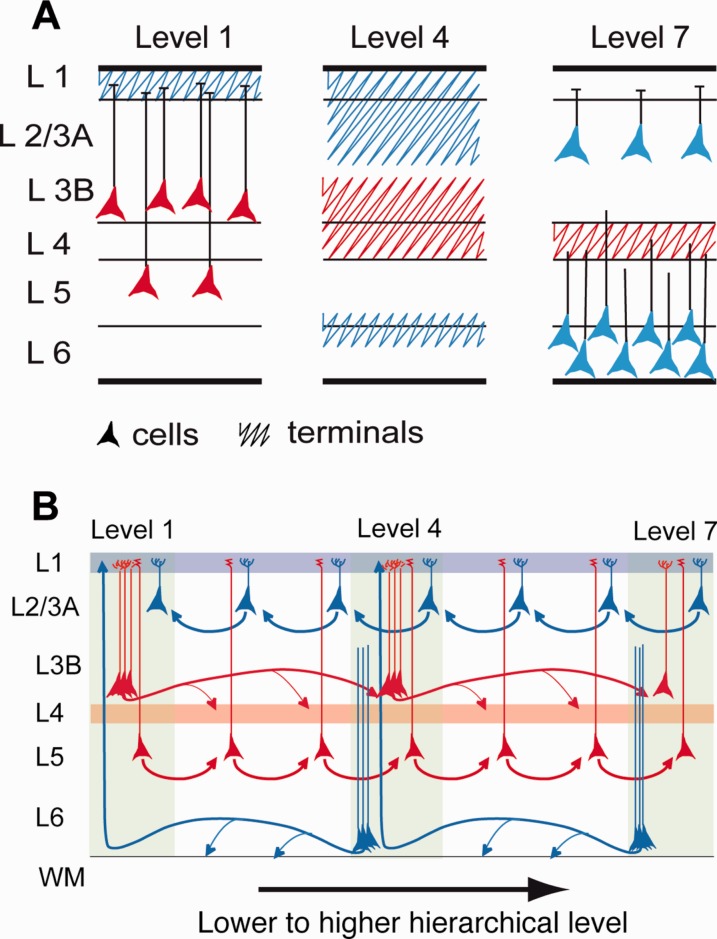
Organization of FB and FF pathways. A: Influence of distance on the distribution of parent neurons (Kennedy and Bullier, 1985; Perkel et al., 1986; Van Essen et al., 1986; Kennedy et al., 1989; Sousa et al., 1991; Rockland et al., 1994; Rockland and Van Hoesen, 1994; Barone et al., 2000). Influence of distance on distribution of terminals (Tigges et al., 1973, 1977; Rockland and Pandya, 1979; Henry et al., 1991; Rockland and Drash, 1996; Anderson and Martin, 2006). FF level 1 neurons project to layer 4 and layer 3B in level 4 and layer 4 of level 7. FB layer 2/3A neurons in level 7 project to layers 1, 2/3A in level 4 and layer 1 of level 1. Level 7 layer 6 FB neurons project to layer 6 in level 4 and layer 1 of level 1. B: Long-distance FF pathway in layer 3B, tightly integrated with layer 4 via basal dendrites and the targeting of layer 4 of upstream areas. Long-distance FB pathway in layer 6 targeting layer 6 of adjacent areas and layer 1 of far-distant downstream areas (Rockland and Van Hoesen, 1994). In the layer 2/3A there is a short-distance FB pathway tightly integrated with layer 1 via apical dendritic tufts. In layer 6 there are two short-distance FF pathways in layer 5 and 6, the layer 5 pathway being in contact with layer 1 via its apical dendrites. The parent populations of FF and FB are highly distinct, and neurons very rarely have FF and FB collaterals.

Figure 2 summarizes the current understanding of the lamination patterns of FF and FB pathways. A number of authors have noted specific distance effects, where an injection of retrograde tracer in low-level areas leads to increasing proportions of supragranular neurons at sequentially higher levels, and inversely injections in high-level areas leads to increasing proportions of infragranular neurons when descending the cortical hierarchy (see legend of Fig. 2 for details). In the back direction, anterograde tracer injection in an area occupying a high position in the cortical hierarchy leads to widely distributed terminals in layers 1, 2, 3, 5, and 6 in areas in the middle areas of the hierarchy whereas, in low areas, terminals are concentrated in layer 1 with only very weak labeling in infragranular layers (Fig. 2A). In the forward direction, anterograde tracer injection in a low area leads to terminals located in layers 3 and 4 in middle areas and layer 4 in higher areas (Fig. 12A).

Taking the present retrograde labeling results together with earlier anterograde studies reveals two sets of pathways in the supra- and infragranular layers (Fig. 2B). In supragranular layers, layer 3B FF neurons are integrated in the underlying input layer (the internal granular layer, layer 4) via their basal dendrites and receive FF input directly from the layer 3B of lower-stream areas and they target layer 4 of high-order areas (Lund et al., 1981). In contrast with layer 3B, layer 2/3A neurons are uniquely integrated in the external granular layer, via their apical dendrites and target downstream areas. In the infragranular layers, layer 6 neurons constitute the major FB pathway, relaying input from neurons in the infragranular layers of upstream areas over short to medium distances, and in addition provide input to layer 1 over long-range distances (Lund et al., 1981; Henry et al., 1991; Angelucci and Bressloff, 2006).

The present results show that the location of FB streams in layer 2/3A and FF streams in 3B is a feature of extrastriate cortex, suggesting a general scheme of FF and FB connections in visual cortex whereby a topologically organized counterstream is located in supragranular layers and a more intermingled set of FF and FB connections constitutes a counterstream in infragranular layers (Fig. 2). All the supragranular FF neurons and some FF neurons in layer 5 had apical dendrites reaching layer 1. FF and FB interareal connections were organized so that parent neurons in the supragranular layers terminated in the homologous layer in their target area, while parent neurons in the infragranular layers terminated in the supra- and infragranular layers, and in particular in layer 1. This compartmentalization of connectivity and the observed location of the apical dendrites lend weight to the suggestion by Rockland that interareal circuits form a system of chains and loops (Rockland, 1994, 1997). For instance, the FF neurons in layer 3B receive FF input largely via their basal dendrites in layers 3B and 4 and form an FF chain. Similarly, the FB neurons in layer 2/3A receive direct FB signals and are part of an FB chain. Interaction between FF and FB streams occurs via loops. Hence, the FF neurons of layers 3B and 5 interact with FB signals via their apical dendrites located in layer 1 and form FF loops. The layer 6 FB neurons interact with FF signals via their apical dendrites in layer 4 and form FB loops.

It has been hypothesized that layer 1 provides a single structure integrating top-down signals and sensory input (Cauller, 1995; Larkum et al., 1999). The major contingent of FB neurons, located in layer 6, has no contact with layer 1, in contrast to the FB layer 2/3A neurons that have apical tufts in layer 1 (Fig. 2B). The infragranular FB neurons, along with the spiny stellate neurons of layer 4, may be better tuned to respond to thalamocortical and corticocortical FF input, whereas layers 2/3 and 5 have been shown to be the only layers that respond to layer 1 (Cauller and Connors, 1994) and show monosynaptic response to FB (Rockland et al., 1994; Johnson and Burkhalter, 1997). These results suggest that modulation of pyramidal neuron activity via FB projections to layer 1 (Cauller, 1995) preferentially influences FF and short-distance FB projections.

### Diffuse versus point-to-point connectivity of FF and FB pathways

The present study used pairs of injections in a single lower or higher-order area to investigate the divergence and convergence of FF and FB pathways. This led to two populations of back-labeled neurons in the source areas, the spatial location of each in the supra- and infragranular layers reflecting the topology of projection from that layer: the greater the divergence-convergence of the projection, the greater the overlap between the two populations and the higher the number of double-labeled neurons in the overlap region. This technique was previously used to explore the topology of corticocortical pathways in adults and during development (Kennedy et al., 1994; Barone et al., 1996; Batardiere et al., 1998).

The topographic precision of supra- and infragranular layer neurons was compared in FF and FB pathways. In both sets of pathways, point-to-point characteristics were sharper in supragranular than infragranular layers. These results extend the suggestion by Salin and Bullier (1995) that it is the distinction between supra- and infragranular layers that largely defines both rates of axonal bifurcation to distant areas and patchiness. It would also seem that topographical precision is defined by the layer rather than by the FF or FB status of the pathway. The present study indicates that supragranular layers exhibit point-to-point connectivity in both FF and FB, and that it is the larger contribution of infragranular layers in the FB pathways, that is responsible for their more diffuse character in comparison of the FF pathways. In support of these findings, note that there is a tendency for the far surround modulation of receptive field response to be more spatially extensive in infragranular than in supragranular layers (Shushruth et al., 2009).

### Distance rules in the visual cortex

SLN has been proposed as an index of hierarchical distance between areas (Barone et al., 2000). In the present study, SLN of pathways interconnecting early visual areas was highly correlated (Fig. 4B). This confirms that the laminar distribution of projection neurons constitutes a powerful regularity in the cortex, which needs to be interpreted by the kind of connectivity diagram shown in Figure 2. The fact that SLN increased with increasing distance reflects the fact that layer 3B is a long-distance FF stream while layer 5/6 is a shorter distance FF stream. The situation is more pronounced in the FB pathways, in which SLN decrease with FB distance reflected the fact that layer 2/3A is a short-distance FB stream, whereas layer 5/6 is a long-distance FB stream. Hence, the FF and FB pathways in extrastriate cortex express combinatorial distance rules.

Physical distance is an important factor to be considered when comparing interareal pathways in the cortical hierarchy. Seventy-five percent of corticocortical neurons in interareal connections are short to medium range. In these pathways, supragranular and infragranular layers are all participating in both FF and FB pathways, each layer contributing its characteristic features to both sets of pathways. It is only the long-distance pathways that show a sharp dichotomy, it is these cases where long-distance FF connections stem uniquely from supragranular layers, exhibit point-to-point connectivity, and target layer 4, in contrast to long-distance FB connections that stem uniquely from infragranular layers, exhibit diffuse connectivity and target layer 1.

### What are the processes supported by the FF and FB counterstreams?

The physiological roles of FF and FB pathways were extensively investigated by silencing a cortical area and examining how this modified activity in its target areas (Girard and Bullier, 1989; Hupe et al., 1998). Globally these reports claimed that FF pathways exert a driving influence on their downstream targets, while FB projections are limited to modulating the response of upstream targets to their driving FF inputs. However, there are some well-documented limitations to this rule. For instance, while inactivation of area V1 was reported to silence area V2 (Girard and Bullier, 1989), after a long-term lesion in area V1 robust visual activity was recorded in area V2 (Schmid et al., 2009), in keeping with the fact that the anatomical input to area V2 includes a moderately strong input from the lateral geniculate nucleus (Markov et al., 2011). More recently, it was reported that silencing V2 induced response facilitation in area V1 via reduction of surround suppression (Nassi et al., 2013). In cat, following inactivation of the thalamic drive, a population of cells in the superficial layers of area V1 continued to respond to visual stimulation, and this visual response was abolished after removal of area V2 (Mignard and Malpeli, 1991). Likewise, FB connections from frontal cortex have been shown to drive memory recall (Tomita et al., 1999). Finally, following inactivation of MT, the visual response of a small number of cells in the superficial layers of area V2 was abolished (Hupe et al., 1998). These and other considerations question the validity of a strict dichotomy between FF as drivers and FB as modulators.

The influence of attention to a visual stimulus has been studied in relation to gamma synchronization in the cortex. Simultaneous recording at corresponding locations in area V4 and the frontal eye field (FEF, subdivided into areas 8L and 8m in the present study) showed that attention led to increased firing rates and enhanced gamma oscillatory coupling between the two areas. This recent study showed that these attention effects occurred significantly earlier in FEF than in V4, suggesting that FEF initiates the coupling between the two areas, and that an increase in LFP gamma power precedes the increase in V4 firing rate (Gregoriou et al., 2009). It was later found that the origin of the attention-linked FEF-V4 gamma coherence was between the FEF supragranular visual-cells, but not the infragranular motor-cells and V4 (Gregoriou et al., 2012). The significance of these results could be far-reaching because recordings in areas V1, V2, and V4 showed strong laminar compartmentalization, with gamma coherence (40–60 Hz) in the supragranular and alpha-beta coherence (6–16 Hz) in the infragranular layers (Buffalo et al., 2011).

These findings show that changes in gamma coherence originate in the FEF, a prefrontal area, and are broadcast over large distances to extrastriate cortex, and subsequently enhance firing rates as part of a top-down attentional effect. This indicates that top-down projections are doing something more than simply regulating sensory input.

It has been hypothesized that the cortex implements a computation in which internal or generative models are used via FB to disambiguate incoming FF signals (Mumford, 1992). A key feature of this theory is the interactive hierarchical computation involving ascending prediction error signals and descending predictions (Lee and Mumford, 2003; Friston, 2010; Markov and Kennedy, 2013). Here we shall address the relevance of the anatomic findings of the present study with respect to these concepts.

The structural asymmetries of the FF and FB pathways and their correspondence to driving and modulatory roles have been cited as a critical feature for hierarchical generative models (Friston, 2003). According to this scheme, FF pathways exhibit sparse axonal bifurcation, are topographically organized and originate in supragranular layers, while FB pathways show abundant bifurcation, diffuse topography, and originate in lower layers. As we have seen, however, these characteristics do not distinguish between FF and FB so much as between infragranular and supragranular pathways. The present results suggest that there is an infragranular and a supragranular component of cortical pathways, be they FF or FB. Because the infragranular component dominates in the FB pathway and the supragranular layer component in the FF, there has been a tendency to characterize FB by its infragranular features and FF by its supragranular features. However, understanding the contribution of both the upper and lower components to both the FF and FB pathways will be a challenge for the development of hierarchical computational models of cortical function. The asymmetry of the FF and FB pathways boils down to their having very different functional roles. Despite the ubiquity of axon bifurcation in the cortex in both FF and FB projections (Kennedy and Bullier, 1985; Bullier and Kennedy, 1987; Nakamura et al., 1993; Rockland and Knutson, 2000; Sincich and Horton, 2003), only on very rare occasions did an FB neuron have an FF collateral; this result is in favor of FF and FB having distinct physiological roles.

The present finding that FF and FB projections in the supragranular layers are highly segregated in ascending and descending streams supports Ullman’s counterstream hypothesis (Ullman, 1995). In the present study, FF and FB streams showed a dual counterstream organization. In Ullman’s counterstream hypothesis, FF and FB connections are highly reciprocal; this we show was not the case: FB pathways covered greater distances (Fig. 7A) and were more numerous (Fig. 7B), so that many FB pathways are not reciprocated by FF pathways (data not shown). In fact, it is these very long-distance FF and FB connections that most closely fit the high asymmetry of Friston’s (2005) model, given that they originate exclusively from FF layers 3B and 6, respectively.

## CONCLUSION

The SLN model of cortical hierarchy emphasizes the specificity of the supragranular layers, which in mammalian evolution undergo an expansion to reach a maximum in primates (Hill and Walsh, 2005; Dehay and Kennedy, 2007; Rakic, 2009). The high-precision counterstream organization in the supragranular layers reported here is generated by a primate-specific germinal zone (Smart et al., 2002; Lukaszewicz et al., 2005). The supra- and infragranular layers are distinguished according to differences in neuronal dynamics (Maier et al., 2010; Sakata and Harris, 2009; Bollimunta et al., 2011) which, along with differences in extrinsic connectivity, suggest a coupling between functional and structural hierarchies (Vezoli et al., 2012).

FF and FB pathways obeyed well-defined distance rules, as reported elsewhere (Ercsey-Ravasz et al., 2013; Markov et al., 2013). The bulk of corticocortical pathways were over short to medium distances, where the pathways ascending and descending the cortical hierarchy are very similar in terms of topographical precision, suggesting highly equivalent capacities. At these short to medium distances, the parent neurons of cortical pathways are both supra- and infragranular, and small differences in topographical precision simply reflect the different proportions of supra- and infragranular layer neurons. Only the long-distance descending projections, originating uniquely from layer 6 and targeting layer 1, would be devoid of supragranular layer connections. Due to their absence of layer 2/3 FB neurons the long-distance FB pathways might have different physiological properties from the shorter-distance FB. Taken together with comparisons of pathway incidence across distance, this suggests that two processing counterstreams are embedded in the cortical hierarchy, and can each be seen as an FF model in terms of control theory. Synaptic zinc has been shown to be characteristic of FB pathways and specific to infragranular layer neurons, layer 2/3 FB being zinc-negative (Ichinohe et al., 2010). Comparing the receptive field properties of layer 2 and 3 neurons will give insight into the functions of the descending pathway of layer 2/3A and the ascending pathway of layer 3B (Shipp et al., 2009). This structure is overlaid by an extensive web of long-distance FB projections that can provide modulatory input to the two counterstreams (Markov et al., 2011, 2012).

The use of continuous scales to estimate the hierarchical organization of the cortex provides an optimized hierarchy with a range of possible values. One could speculate that the functional hierarchy is dynamic and task-dependent, with the structural hierarchy providing boundary values.

It will be important to extend the present quantitative analysis of retrograde tracers by molecular characterization of the parent neurons (Hof and Morrison, 1995; Yamamori, 2011; Bernard et al., 2012). A necessary and complementary development will be to use anterograde tracers to examine the laminar integration of interareal connectivity, combining quantification and morphological characterization at the synaptic level (Anderson et al., 1998; Wang et al., 2012).
